# Supplementary material for: Measurement of changes to the menstrual cycle: A transdisciplinary systematic review evaluating measure quality and utility for clinical trials
Source: PLoS One. 2024 Jul 25;19(7):e0306491. doi: 10.1371/journal.pone.0306491 (PMC11271926; doi:10.1371/journal.pone.0306491)
Supplement: S2 Table — (PDF) [file pone.0306491.s002.pdf]

S2 Table. All articles included after title/abstract screening and full text review.

Measures being evaluated/validated	First author	Year	Region	Country	Study Design	Ref.
**Articles reporting on validating/evaluating full instruments (n=133)**						
Aberdeen Menorrhagia Severity Scale	Ruta	1995	Europe	United Kingdom	Cross-Sectional	[1]
Aberdeen Menorrhagia Severity Scale	Abu-Rafea	2012	Middle East	Saudi Arabia	Cross-Sectional	[2]
Adolescent Dysmenorrhic Self-Care Scale	Ching-Hsing	2004	Asia	Taiwan	Cross-Sectional	[3]
Adolescent Dysmenorrhic Self-Care Scale	Wong	2013	Asia	Hong Kong	Cross-Sectional	[4]
Alkaline Hematin Assay	van Eijkeren	1986	Europe	Netherlands	Prospective Cohort	[5]
Bleeding and Pelvic Discomfort Scale	Hudgens	2022	Multiple	Multiple	Randomized Controlled Trial	[6]
Daily diary, menopause classification	Paramsothy	2013	North America	United States	Prospective Cohort	[7]
Daily diary, menstrual cycle length	Johannes	2000	North America	United States	Prospective Cohort	[8]
Dysmenorrhea Daily Diary	Nguyen	2015	North America	United States	Cross-Sectional	[9]
Dysmenorrhea Daily Diary	Nguyen	2017	Multiple	Multiple	Cross-Sectional	[10]
Dysmenorrhea Symptom Interference Scale	Chen	2021	North America	United States	Mixed methods	[11]
electronic Personal Assessment Questionnaire - Menstrual, Pain, and Hormonal	Gray	2019	Europe	United Kingdom	Cross-Sectional	[12]
Endometriosis Daily Diary	Guan	2022	North America	United States	Prospective Cohort	[13]
Endometriosis Daily Pain Impact Diary	Wyrwich	2018	North America	United States	Prospective Cohort	[14]
Endometriosis Health Profile-30	Jones	2001	Europe	United Kingdom	Cross-Sectional	[15]
Endometriosis Health Profile-30	Mengarda	2008	South America	Brazil	Cross-Sectional	[16]

Measures being evaluated/validated	First author	Year	Region	Country	Study Design	Ref.
Endometriosis Health Profile-30	Grundstrom	2020	Europe	Sweden	Cross-Sectional	[17]
Endometriosis Health Profile-30	Grundstrom	2020	Europe	Sweden	Cross-Sectional	[18]
Endometriosis Health Profile-30	Jones	2004	Europe	United Kingdom	Prospective Cohort	[19]
Endometriosis Health Profile-30	Jenkinson	2008	North America	United States	Cross-Sectional	[20]
Endometriosis Health Profile-30	Hansen	2022	Europe	Denmark	Cross-Sectional	[21]
Endometriosis Health Profile-30	Khong	2010	Oceania	Australia	Cross-Sectional	[22]
Endometriosis Health Profile-30	Verket	2018	Europe	Norway	Cross-Sectional	[23]
Endometriosis Health Profile-30	Jones	2006	Europe	United Kingdom	Cross-Sectional	[24]
Endometriosis Health Profile-30	van de Burgt	2011	Europe	Netherlands	Case Control	[25]
Endometriosis Health Profile-30	Van de Burgt	2013	Europe	Netherlands	Prospective Cohort	[26]
Endometriosis Health Profile-30	Wickstrom	2017	Europe	Sweden	Prospective Cohort	[27]
Endometriosis Health Profile-30	Nojomi	2011	Middle East	Iran	Cross-Sectional	[28]
Endometriosis Health Profile-30	Mansor	2023	Asia	Malaysia	Cross-Sectional	[29]
Endometriosis Health Profile-30	Maiorana	2012	Europe	Italy	Cross-Sectional	[30]
Endometriosis Health Profile-30	Marí-Alexandre	2022	Europe	Spain	Cross-Sectional	[31]
Endometriosis Health Profile-30	Nogueria-Silva	2015	Europe	Portugal	Cross-Sectional	[32]
Endometriosis Health Profile-30	Chauvet	2017	Europe	France	Cross-Sectional	[33]
Endometriosis Health Profile-30	Jia	2013	Asia	China	Cross-Sectional	[34]
Endometriosis Health Profile-5	Jones	2004	Europe	United Kingdom	Cross-Sectional	[35]

Measures being evaluated/validated	First author	Year	Region	Country	Study Design	Ref.
Endometriosis Health Profile-5	Fauconnier	2017	Europe	France	Case Control	[36]
Endometriosis Health Profile-5	Aubry	2017	Europe	France	Prospective Cohort	[37]
Endometriosis Health Profile-5	Mikuš	2023	Europe	Croatia	Prospective Cohort	[38]
Endometriosis Health Profile-5	Selcuk	2015	Europe	Turkey	Case Control	[39]
Endometriosis Impact Questionnaire	Moradi	2019	Oceania	Australia	Cross-Sectional	[40]
Endometriosis Impact Scale & Endometriosis Symptom Diary	Gater	2020	Multiple	Multiple	Mixed methods	[41]
Endometriosis Pain and Bleeding Diary	Deal	2010	North America	United States	Prospective Cohort	[42]
Endometriosis Pain Daily Diary	Van Nooten	2018	Multiple	Multiple	Mixed methods	[43]
Endometriosis Reproductive Health Questionnaire	Namazi	2021	Middle East	Iran	Mixed methods	[44]
Endometriosis Self-Assessment Tool	Cho	2022	Asia	South Korea	Cross-Sectional	[45]
Endometriosis Treatment Satisfaction Questionnaire	Deal	2010	North America	United States	Randomized Controlled Trial	[46]
ENDOPAIN-4D	Fauconnier	2018	Europe	France	Mixed methods	[47]
ENDOPAIN-4D	Puchar	2021	Europe	France	Prospective Cohort	[48]
ENDOPAIN-4D	Ahmadpour	2022	Middle East	Iran	Cross-Sectional	[49]
EndoWheel	As-Sanie	2021	North America	United States	Mixed methods	[50]
Fibroid Symptom Diary	Deal	2011	North America	United States	Mixed methods	[51]
Functional and Emotional Measure of Dysmenorrhea	Li	2012	Asia	China	Cross-Sectional	[52]
Injustice Experience Questionnaire-Chronic and the Contribution of Perceived Injustice	Yamada	2019	Asia	Japan	Prospective Cohort	[53]

Measures being evaluated/validated	First author	Year	Region	Country	Study Design	Ref.
Mansfield-Voda-Jorgensen Menstrual Bleeding Scale	Mansfield	2004	North America	United States	Prospective Cohort	[54]
Measure compilation (Olliges)	Olliges	2021	Europe	Germany	Case Control	[55]
Menorrhagia Impact Questionnaire	Bushnell	2010	North America	United States	Case Control	[56]
(Menorrhagia) Multi-Attribute Utility Score	Shaw	1998	Europe	United Kingdom	Mixed methods	[57]
(Menorrhagia) Multi-Attribute Utility Score	Habiba	2010	Europe	United Kingdom	Prospective Cohort	[58]
(Menorrhagia) Multi-Attribute Utility Score	Pattison	2011	Europe	United Kingdom	Randomized Controlled Trial	[59]
Menstrual Attitudes Questionnaire	Brooks-Gunn	1980	North America	United States	Cross-Sectional	[60]
Menstrual Attitudes Questionnaire	Bramwell	2002	Europe; Asia	United Kingdom; India	Cross-Sectional	[61]
Menstrual Attitudes Questionnaire	Firat	2009	Europe; Asia	Turkey	Cross-Sectional	[62]
Menstrual Attitudes Questionnaire	Bargiota	2016	Europe	Greece	Cross-Sectional	[63]
Menstrual Attitudes Questionnaire	Kawata	2022	Asia	Nepal	Cross-Sectional	[64]
Menstrual Bleeding Questionnaire	Rezende	2023	South America	Brazil	Prospective Cohort	[65]
Menstrual Bleeding Questionnaire	Matteson	2015	North America	United States	Prospective Cohort; Cross-Sectional	[66]
Menstrual Bleeding Questionnaire	Pike	2021	North America	Canada	Cross-Sectional, Prospective Cohort	[67]
Menstrual Bleeding Questionnaire	Rodpetch	2021	Asia	Thailand	Cross-Sectional	[68]
Menstrual Blood Loss Score Questionnaire	Toxqui	2014	Europe	Spain	Prospective Cohort	[69]
Menstrual Collection	Chimbira	1980	Europe	United Kingdom	Prospective Cohort	[70]

Measures being evaluated/validated	First author	Year	Region	Country	Study Design	Ref.
Menstrual Collection	Gleeson	1993	Europe	Ireland	Prospective Cohort	[71]
Menstrual Collection	Gannon	1996	Europe	United Kingdom	Prospective Cohort	[72]
Menstrual Collection	Fraser	2001	Oceania	Australia	Prospective Cohort	[73]
Menstrual Collection	Gudmundsdottir	2009	Europe	Iceland	Prospective Cohort	[74]
Menstrual Distress Questionnaire (Moos)	Moos	1968	North America	United States	Cross-Sectional	[75]
Menstrual Distress Questionnaire (Moos)	Lee	2009	Asia	China	Cross-Sectional	[76]
Menstrual Distress Questionnaire (Vannuccini)	Vannuccini	2021	Europe	Italy	Cross-Sectional	[77]
Menstrual Distress Questionnaire (Vannuccini)	Cassioli	2023	Europe; Oceania	Multiple	Cross-Sectional	[78]
Menstrual Health Instrument	Shin	2018	Asia	South Korea	Mixed methods	[79]
Menstrual Health Seeking Behaviors Questionnaire	Darabi	2018	Middle East	Iran	Cross-Sectional	[80]
Menstrual Hygiene Management Scale	Ramaiya	2020	Asia	India	Mixed methods	[81]
Menstrual Insecurity Tool	Caruso	2020	Asia	India	Mixed methods	[82]
Menstrual Joy Questionnaire	Aubeeluck	2002	Europe	United Kingdom	Cross-Sectional	[83]
Menstrual Practices Questionnaire	Hennegan	2020	Africa	Uganda	Cross-Sectional	[84]
Menstrual Record and Recall	Heath	1999	Oceania	New Zealand	Cross-Sectional	[85]
Menstrual Self-Evaluation Scale	Roberts	2004	North America	United States	Cross-Sectional	[86]
Menstruation-Related Activity Restrictions Questionnaire	Garg	2021	Asia	India	Cross-Sectional	[87]
Military Women's Attitudes Toward Menstrual Suppression Scale	Trego	2009	North America	United States	Mixed methods	[88]

Measures being evaluated/validated	First author	Year	Region	Country	Study Design	Ref.
New Zealand Survey of Adolescent Girls' Menstruation	Farquhar	2009	Oceania	New Zealand	Cross-Sectional	[89]
Numeric Rating Scale	deArruda	2022	South America	Brazil	Prospective Cohort	[90]
Numeric Rating Scale	Pokrzywinski	2021	North America	Multiple	Randomized Controlled Trial	[91]
Pain Drawing	Rodrigues	2022	South America	Brazil	Cross-Sectional	[92]
Period ImPact and Pain Assessment	Parker	2022	Oceania	Australia	Cross-Sectional	[93]
PERIOD-QOL	Lancastle	2023	Europe	United Kingdom	Cross-Sectional	[94]
Pictorial Blood Loss Assessment Charts & Menstrual Pictograms	Higham	1990	Europe	United Kingdom	Cross-Sectional	[95]
Pictorial Blood Loss Assessment Charts & Menstrual Pictograms	Janssen	1995	Europe	Netherlands	Cross-Sectional	[96]
Pictorial Blood Loss Assessment Charts & Menstrual Pictograms	Barr	1999	Africa	Nigeria	Cross-Sectional	[97]
Pictorial Blood Loss Assessment Charts & Menstrual Pictograms	Reid	2000	Europe	United Kingdom	Prospective Cohort	[98]
Pictorial Blood Loss Assessment Charts & Menstrual Pictograms	Wyatt	2001	Europe	United Kingdom	Prospective Cohort	[99]
Pictorial Blood Loss Assessment Charts & Menstrual Pictograms	Sanchez	2012	North America	United States	Prospective Cohort	[100]
Pictorial Blood Loss Assessment Charts & Menstrual Pictograms	Larsen	2013	North America	United States	Randomized Controlled Trial	[101]
Pictorial Blood Loss Assessment Charts & Menstrual Pictograms	Magnay	2013	Europe	United Kingdom	Laboratory	[102]
Pictorial Blood Loss Assessment Charts & Menstrual Pictograms	Magnay	2014	Europe	United Kingdom	Prospective Cohort	[103]
Pictorial Blood Loss Assessment Charts & Menstrual Pictograms	Hald	2014	Europe	Norway	Retrospective Cohort	[104]
Pictorial Blood Loss Assessment Charts & Menstrual Pictograms & Uterine Fibroid Daily Bleeding Diary	Haberland	2020	Europe	Germany	Randomized Controlled Trial	[105]

Measures being evaluated/validated	First author	Year	Region	Country	Study Design	Ref.
Prospective self report, menstrual regularity	Weller	1998	Middle East	Israel	Prospective Cohort	[106]
Quantitative model for menstrual blood loss	Schumacher	2012	Multiple	Multiple	Retrospective Cohort	[107]
Retrospective self-report, last menstrual period	Wegienka	2005	North America	United States	Prospective Cohort	[108]
Retrospective self report, menstrual discomfort	Jukic	2008	North America	United States	Prospective Cohort	[109]
Retrospective self report, menstrual length (Bachand)	Bachand	2009	North America	United States	Prospective Cohort	[110]
Retrospective self report, menstrual length (Small & Jukic)	Jukic	2007	North America	United States	Prospective Cohort	[111]
Retrospective self report, menstrual length (Small & Jukic)	Small	2007	North America	United States	Prospective Cohort	[112]
SAMANTA Questionnaire	Calaf	2020	Europe	Spain	Cross-Sectional	[113]
SAMANTA Questionnaire	Perelló-Capo	2023	Europe	Spain	Prospective Cohort	[114]
Spanish Society of Contraception Quality-of-Life	Pérez-Campos	2011	Europe	Spain	Prospective Cohort	[115]
Squeezing Pain Bulb	Kantarovich	2021	North America	United States	Cross-Sectional	[116]
Uterine Fibroid Symptom and Quality of Life Questionnaire	Spies	2002	North America	United States	Cross-Sectional	[117]
Uterine Fibroid Symptom and Quality of Life Questionnaire	Harding	2008	North America	United States	Prospective Cohort	[118]
Uterine Fibroid Symptom and Quality of Life Questionnaire	Silva	2016	South America	Brazil	Case Control	[119]
Uterine Fibroid Symptom and Quality of Life Questionnaire	Oliveira Brito	2017	South America	Brazil	Cross-Sectional	[120]
Uterine Fibroid Symptom and Quality of Life Questionnaire	Coyne	2017	North America	United States	Randomized Controlled Trial	[121]
Uterine Fibroid Symptom and Quality of Life Questionnaire	Coyne	2019	North America	Multiple	Randomized Controlled Trial	[122]
Uterine Fibroid Symptom and Quality of Life Questionnaire	Yeung	2019	Asia	Hong Kong	Cross-Sectional	[123]

Measures being evaluated/validated	First author	Year	Region	Country	Study Design	Ref.
Uterine Fibroid Symptom and Quality of Life Questionnaire	Calaf	2020	Europe	Spain	Cross-Sectional	[124]
Uterine Fibroid Symptom and Quality of Life Questionnaire	Keizer	2021	Europe	Netherlands	Cross-Sectional	[125]
Visual Analogue Scales: Pain	Gerlinger	2012	Europe	Germany	Retrospective Cohort	[126]
Visual Analogue Scales: Pain	Gerlinger	2010	Europe	Multiple	Retrospective Cohort	[127]
Visual Analogue Scales: Bleeding	Perelló	2022	Europe	Spain	Retrospective Cohort	[128]
World Health Organization Disability Assessment Schedule 2.0	deArruda	2023	South America	Brazil	Cross-Sectional	[129]
Working Ability, Location, Intensity, Days of Pain, Dysmenorrhea Score	Teherán	2018	South America	Colombia	Cross-Sectional	[130]
Working Stressors and Coping Strategies Associated with Menstrual Symptoms Among Nurses	Yu	2021	Asia	Taiwan	Cross-Sectional	[131]
World Endometriosis Research Foundation Endometriosis Phenome and Biobanking Harmonisation Project Standard Questionnaire	Vitonis	2014	Multiple	Multiple	Other	[132]
World Endometriosis Research Foundation Endometriosis Phenome and Biobanking Harmonisation Project Standard Questionnaire	Dimentberg	2021	North America	Canada	Prospective Cohort	[133]
**Articles reporting on validating/evaluating applicable subscales (n=19)**						
Low Energy Availability in Females Questionnaire	Melin	2014	Europe	Multiple	Prospective Cohort	[134]
Low Energy Availability in Females Questionnaire	deMaria	2021	South America	Brazil	Prospective Cohort	[135]
Menstrual Cycle-Related Signs and Symptoms Questionnaire	Sutthibut	2021	Asia	Thailand	Cross-Sectional	[136]
Menstrual Symptom Questionnaire	Chesney	1975	North America	United States	Cross-Sectional	[137]
Menstrual Symptom Questionnaire	Nelson	1984	North America	United States	Cross-Sectional	[138]
Menstrual Symptom Questionnaire	Negriff	2009	North America	United States	Cross-Sectional	[139]

Measures being evaluated/validated	First author	Year	Region	Country	Study Design	Ref.
Midlife Women's Symptom Index	Im	2006	North America	United States	Cross-Sectional	[140]
ORTHO Birth Control Satisfaction Assessment Tool	Mathias	2006	North America	United States	Mixed methods	[141]
ORTHO Birth Control Satisfaction Assessment Tool	Colwell	2006	North America	United States	Cross-Sectional	[142]
Ovulation and Menstruation Health Questionnaire	Mahalingaiah	2020	North America	United States	Mixed methods	[143]
Polycystic Ovary Syndrome Quality of Life Scale	Saei Ghare Naz	2023	Middle East	Iran	Mixed methods	[144]
Polycystic Ovary Syndrome Quality of Life Scale	Cronin	1998	North America	United States	Mixed methods	[145]
Polycystic Ovary Syndrome Quality of Life Scale	Jones	2004	Europe	United Kingdom	Cross-Sectional	[146]
Polycystic Ovary Syndrome Quality of Life Scale	Guyatt	2004	Europe	United Kingdom	Randomized Controlled Trial	[147]
Polycystic Ovary Syndrome Quality of Life Scale	Jedel	2008	Europe	Sweden	Randomized Controlled Trial	[148]
Polycystic Ovary Syndrome Quality of Life Scale	Bazarganipour	2012	Middle East	Iran	Cross-Sectional	[149]
Polycystic Ovary Syndrome Quality of Life Scale	Bazarganipour	2013	Middle East	Iran	Cross-Sectional	[150]
Polycystic Ovary Syndrome Quality of Life Scale	Chung	2016	Asia	China	Cross-Sectional	[151]
Women Shift Workers Reproductive Health Questionnaire	Nikpour	2020	Middle East	Iran	Mixed methods	[152]
**Articles reporting on validating/evaluating instruments with applicable items/questions (n=14)**						
Adolescent Menstrual Attitude Questionnaire	Morse	1993	North America	Canada	Not assessed	[153]
Adolescent Menstrual Attitude Questionnaire	Morse	1993	North America	Canada	Not assessed	[154]
CARDIA Women's Reproductive Health Questionnaire	Whitham	2013	North America	United States	Not assessed	[155]

Measures being evaluated/validated	First author	Year	Region	Country	Study Design	Ref.
Clinical Tool for Diagnosis of PCOS	Pedersen	2007	North America	Canada	Not assessed	[156]
Clinically Validated Scores for Endometriosis Diagnosis	Chapron	2022	Europe	France	Not assessed	[157]
Experience Sampling Method	van Barneveld	2023	Europe	Netherlands	Not assessed	[158]
Immune Thrombocytopenic Purpura Patient Assessment Questionnaire	Mathias	2007	Multiple	Multiple	Not assessed	[159]
Painful Periods Screening Tool	DiBenedetti	2018	North America	United States	Not assessed	[160]
Polycystic Ovary Syndrome Questionnaire-50	Nasiri-Amiri	2016	Middle East	Iran	Not assessed	[161]
Polycystic Ovary Syndrome Questionnaire-50	Stevanovic	2019	Europe	Serbia	Not assessed	[162]
Self-Efficacy in Addressing Menstrual Needs Scale	Hunter	2022	Asia	Bangladesh	Not assessed	[163]
Stellenbosch Endometriosis Quality of Life Measure	Rizwana	2018	Africa	South Africa	Not assessed	[164]
Structured Endometriosis Questionnaire	Hackethal	2011	Europe	Germany	Not assessed	[165]
The International Spinal Cord Injury Female Sexual and Reproductive Function	Alexander	2011	Multiple	Multiple	Not assessed	[166]
**Articles reporting on validating/evaluating general instruments in menstruating populations (n=8)**						
Health Related Productivity Questionnaire	Pokrzywinski	2020	Multiple	Multiple	Not assessed	[167]
Patient-Generated Index of Quality of Life	Ruta	1999	Europe	United Kingdom	Not assessed	[168]
Patient-Reported Outcomes Measurement Information System	Schneider	2013	North America	United States	Not assessed	[169]
Patient-Reported Outcomes Measurement Information System	Pokrzywinski	2020	North America	Multiple	Not assessed	[170]
Survey of Pain Attitudes	Ferreira-Valente	2019	Europe	Portugal	Not assessed	[171]
Women's Health Questionnaire	Hunter	1992	Europe	United Kingdom	Not assessed	[172]

Measures being evaluated/validated	First author	Year	Region	Country	Study Design	Ref.
Women's Health Questionnaire	Hunter	2000	Europe	United Kingdom	Not assessed	[173]
Women's Health Questionnaire	Colantonio	2011	North America	Canada	Not assessed	[174]

References

1. Ruta DA, Garratt AM, Chadha YC, Flett GM, Hall MH, Russell IT. Assessment of patients with menorrhagia: How valid is a structured clinical history as a measure of health status? Quality of Life Research. 1995;4: 33–40. doi:10.1007/BF00434381

2. Abu-Rafea BF, Vilos GA, Al Jasser RS, Al Anazy RM, Javaid K, Al-Mandeel HM. Linguistic and clinical validation of the Arabic-translated Aberdeen Menorrhagia Severity Scale as an indicator of quality of life for women with abnormal uterine bleeding. Saudi Med J. 2012;33: 869–74. Available: http://www.ncbi.nlm.nih.gov/pubmed/22886120

3. Ching-Hsing H, Meei-Ling G, Hsin-Chun M, Chung-Yi L. The development and psychometric testing of a self-care scale for dysmenorrhic adolescents. The journal of nursing research. 2004;12: 119–30. doi:10.1097/01.jnr.0000387495.01557.aa

4. Wong CL, Ip WY, Choi KC, Shiu TY. Translation and validation of the Chinese-Cantonese version of the Adolescent Dysmenorrhic Self-Care Scale in Hong Kong adolescent girls. J Clin Nurs. 2013;22: 1510–1520. doi:10.1111/jocn.12019

5. van Eijkeren MA, Scholten PC, Christiaens GC, Alsbach GP, Haspels AA. The alkaline hematin method for measuring menstrual blood loss--a modification and its clinical use in menorrhagia. Eur J Obstet Gynecol Reprod Biol. 1986;22: 345–51. doi:10.1016/0028-2243(86)90124-3

6. Hudgens S, Gauthier M, Hunsche E, Kang J, Li Y, Scippa K, et al. Development of the Bleeding and Pelvic Discomfort Scale for Use in Women With Heavy Menstrual Bleeding Associated With Uterine Fibroids. Value in Health. 2022;25. doi:10.1016/j.jval.2022.06.005

7. Paramsothy P, Harlow SD, Elliott MR, Lisabeth LD, Crawford SL, Randolph JF. Classifying menopause stage by menstrual calendars and annual interviews: need for improved questionnaires. Menopause. 2013;20: 727–35. doi:10.1097/GME.0b013e3182825ff2

8. Johannes CB, Crawford SL, Woods J, Goldstein RB, Tran D, Mehrotra S, et al. An Electronic Menstrual Cycle Calendar: Comparison of Data Quality With a Paper Version. Menopause. 2000;7: 200–208. doi:10.1097/00042192-200007030-00011

9. Nguyen AM, Humphrey L, Kitchen H, Rehman T, Norquist JM. A qualitative study to develop a patient-reported outcome for dysmenorrhea. Quality of Life Research. 2015;24: 181–191. doi:10.1007/s11136-014-0755-z

10. Nguyen AM, Arbuckle R, Korver T, Chen F, Taylor B, Turnbull A, et al. Psychometric validation of the dysmenorrhea daily diary (DysDD): a patient-reported outcome for dysmenorrhea. Quality of Life Research. 2017;26: 2041–2055. doi:10.1007/s11136-017-1562-0

11. Chen CX, Murphy T, Ofner S, Yahng L, Krombach P, LaPradd M, et al. Development and Testing of the Dysmenorrhea Symptom Interference (DSI) Scale. West J Nurs Res. 2021;43: 364–373. doi:10.1177/0193945920942252

12. Gray TG, Moores KL, James E, Connor ME, Jones GL, Radley SC. Development and initial validation of an electronic personal assessment questionnaire for menstrual, pelvic pain and

gynaecological hormonal disorders (ePAQ-MPH). European Journal of Obstetrics & Gynecology and Reproductive Biology. 2019;238: 148–156. doi:10.1016/j.ejogrb.2019.05.024

13. Guan Y, Nguyen AM, Wratten S, Randhawa S, Weaver J, Arbelaez F, et al. The endometriosis daily diary: qualitative research to explore the patient experience of endometriosis and inform the development of a patient-reported outcome (PRO) for endometriosis-related pain. J Patient Rep Outcomes. 2022;6: 5. doi:10.1186/s41687-021-00409-8

14. Wyrwich KW, O'Brien CF, Soliman AM, Chwalisz K. Development and Validation of the Endometriosis Daily Pain Impact Diary Items to Assess Dysmenorrhea and Nonmenstrual Pelvic Pain. Reproductive Sciences. 2018;25: 1567–1576. doi:10.1177/1933719118789509

15. Jones G, Kennedy S, Barnard A, Wong J, Jenkinson C. Development of an endometriosis quality-of-life instrument: The Endometriosis Health Profile-30. Obstetrics and gynecology. 2001;98: 258–64. doi:10.1016/s0029-7844(01)01433-8

16. Mengarda CV, Passos EP, Picon P, Costa AF, Picon PD. Validação de versão para o português de questionário sobre qualidade de vida para mulher com endometriose (Endometriosis Health Profile Questionnaire - EHP-30). Revista Brasileira de Ginecologia e Obstetrícia. 2008;30: 384–392. doi:10.1590/S0100-72032008000800003

17. Grundström H, Rauden A, Olovsson M. Cross-cultural adaptation of the Swedish version of Endometriosis Health Profile-30. J Obstet Gynaecol (Lahore). 2020;40: 969–973. doi:10.1080/01443615.2019.1676215

18. Grundström H, Rauden A, Wikman P, Olovsson M. Psychometric evaluation of the Swedish version of the 30-item endometriosis health profile (EHP-30). BMC Womens Health. 2020;20: 204. doi:10.1186/s12905-020-01067-6

19. Jones G, Jenkinson C, Kennedy S. Evaluating the responsiveness of the endometriosis health profile questionnaire: The EHP-30. Quality of Life Research. 2004;13: 705–713. doi:10.1023/B:QURE.0000021316.79349.af

20. Jenkinson C, Kennedy S, Jones G. Evaluation of the American version of the 30-item Endometriosis Health Profile (EHP-30). Quality of Life Research. 2008;17: 1147–1152. doi:10.1007/s11136-008-9403-9

21. Hansen KE, Lambek R, Røssaak K, Egekvist AG, Marschall H, Forman A, et al. Health-related quality of life in women with endometriosis: psychometric validation of the Endometriosis Health Profile 30 questionnaire using confirmatory factor analysis. Hum Reprod Open. 2022;2022. doi:10.1093/hropen/hoab042

22. Khong S-Y, Lam A, Luscombe G. Is the 30-item Endometriosis Health Profile (EHP-30) suitable as a self-report health status instrument for clinical trials? Fertil Steril. 2010;94: 1928–1932. doi:10.1016/j.fertnstert.2010.01.047

23. Verket NJ, Andersen MH, Sandvik L, Tanbo TG, Qvigstad E. Lack of cross-cultural validity of the Endometriosis Health Profile-30. J Endometr Pelvic Pain Disord. 2018;10: 107–115. doi:10.1177/2284026518780638

24. Jones G, Jenkinson C, Taylor N, Mills A, Kennedy S. Measuring quality of life in women with endometriosis: tests of data quality, score reliability, response rate and scaling assumptions of the Endometriosis Health Profile Questionnaire. Human Reproduction. 2006;21: 2686–2693. doi:10.1093/humrep/del231

25. van de Burgt TJM, Hendriks JCM, Kluivers KB. Quality of life in endometriosis: evaluation of the Dutch-version Endometriosis Health Profile–30 (EHP-30). Fertil Steril. 2011;95: 1863–1865. doi:10.1016/j.fertnstert.2010.11.009

26. van de Burgt TJM, Kluivers KB, Hendriks JCM. Responsiveness of the Dutch Endometriosis Health Profile-30 (EHP-30) questionnaire. European Journal of Obstetrics & Gynecology and Reproductive Biology. 2013;168: 92–94. doi:10.1016/j.ejogrb.2012.12.037

27. Wickström KW, Spira J, Edelstam G. Responsiveness of the Endometriosis Health Profile-30 questionnaire in a Swedish sample: an observational study. Clin Exp Obstet Gynecol. 2017;44: 413–418. doi:10.12891/ceog3599.2017

28. Nojomi M, Bijari B, Akhbari R, Kashanian M. The Assessment of Reliability and Validity of Persian Version of the Endometriosis Health Profile (EHP-30). Iran J Med Sci. 2011;36: 84–9. Available: http://www.ncbi.nlm.nih.gov/pubmed/23358588

29. Mansor M, Chong MC, Chui PL, Hamdan M, Basha MAMK. The psychometric properties test of the Malay version of the endometriosis health profile-30. Saudi Med J. 2023;44. doi:10.15537/smj.2023.44.9.20230228

30. Maiorana A, Scafidi Fonti GM, Audino P, Rosini R, Alio L, Oliveri AM, et al. The role of EHP-30 as specific instrument to assess the quality of life of Italian women with endometriosis. Minerva Ginecol. 2012;64: 231–8. Available: http://www.ncbi.nlm.nih.gov/pubmed/22635018

31. Marí-Alexandre J, García-Oms J, Agababyan C, Belda-Montesinos R, Royo-Bolea S, Varo-Gómez B, et al. Toward an improved assessment of quality of life in endometriosis: evaluation of the Spanish version of the Endometriosis Health Profile 30. Journal of Psychosomatic Obstetrics and Gynecology. 2022;43. doi:10.1080/0167482X.2020.1795827

32. Nogueira-Silva C, Costa P, Martins C, Barata S, Alho C, Calhaz-Jorge C, et al. Validação da Versão Portuguesa do Questionário EHP-30 (The Endometriosis Health Profile-30). Acta Med Port. 2015;28: 347–356. doi:10.20344/amp.5778

33. Chauvet P, Auclair C, Mourgues C, Canis M, Gerbaud L, Bourdel N. Psychometric properties of the French version of the Endometriosis Health Profile-30, a health-related quality of life instrument. J Gynecol Obstet Hum Reprod. 2017;46: 235–242. doi:10.1016/j.jogoh.2017.02.004

34. Jia S-Z, Leng J-H, Sun P-R, Lang J-H. Translation and psychometric evaluation of the simplified Chinese-version Endometriosis Health Profile-30. Human Reproduction. 2013;28: 691–697. doi:10.1093/humrep/des426

35. Jones G, Jenkinson C, Kennedy S. Development of the Short Form Endometriosis Health Profile Questionnaire: the EHP-5. Qual Life Res. 2004;13: 695–704. doi:10.1023/B:QURE.0000021321.48041.0e

36. Fauconnier A, Huchon C, Chaillou L, Aubry G, Renouvel F, Panel P. Development of a French version of the Endometriosis Health Profile 5 (EHP-5): cross-cultural adaptation and psychometric evaluation. Quality of Life Research. 2017;26: 213–220. doi:10.1007/s11136-016-1346-y

37. Aubry G, Panel P, Thiollier G, Huchon C, Fauconnier A. Measuring health-related quality of life in women with endometriosis: comparing the clinimetric properties of the Endometriosis Health Profile-5 (EHP-5) and the EuroQol-5D (EQ-5D). Human Reproduction. 2017;32: 1258–1269. doi:10.1093/humrep/dex057

38. Mikuš M, Matak L, Vujić G, Škegro B, Škegro I, Augustin G, et al. The short form endometriosis health profile questionnaire (EHP-5): psychometric validity assessment of a Croatian version. Arch Gynecol Obstet. 2023;307: 87–92. doi:10.1007/s00404-022-06691-1

39. Selcuk S, Sahin S, Demirci O, Aksoy B, Eroglu M, Ay P, et al. Translation and validation of the Endometriosis Health Profile (EHP-5) in patients with laparoscopically diagnosed endometriosis. European Journal of Obstetrics & Gynecology and Reproductive Biology. 2015;185: 41–44. doi:10.1016/j.ejogrb.2014.11.039

40. Moradi M, Parker M, Sneddon A, Lopez V, Ellwood D. The Endometriosis Impact Questionnaire (EIQ): a tool to measure the long-term impact of endometriosis on different aspects of women's lives. BMC Womens Health. 2019;19: 64. doi:10.1186/s12905-019-0762-x

41. Gater A, Taylor F, Seitz C, Gerlinger C, Wichmann K, Haberland C. Development and content validation of two new patient-reported outcome measures for endometriosis: the Endometriosis Symptom Diary (ESD) and Endometriosis Impact Scale (EIS). J Patient Rep Outcomes. 2020;4: 13. doi:10.1186/s41687-020-0177-3

42. Deal LS, DiBenedetti D, Williams VS, Fehnel SE. The development and validation of the daily electronic Endometriosis Pain and Bleeding Diary. Health Qual Life Outcomes. 2010;8: 64. doi:10.1186/1477-7525-8-64

43. van Nooten FE, Cline J, Elash CA, Paty J, Reaney M. Development and content validation of a patient-reported endometriosis pain daily diary. Health Qual Life Outcomes. 2018;16: 3. doi:10.1186/s12955-017-0819-1

44. Namazi M, Zareiyan A, Jafarabadi M, Behboodi Moghadam Z. Endometriosis reproductive health questionnaire (ERHQ): A self-administered questionnaire to measure the reproductive health in women with endometriosis. J Gynecol Obstet Hum Reprod. 2021;50: 101860. doi:10.1016/j.jogoh.2020.101860

45. Cho H-H, Yoon Y-S. Development of an endometriosis self-assessment tool for patient. Obstet Gynecol Sci. 2022;65: 256–265. doi:10.5468/ogs.21252

46. Deal LS, Williams VSL, DiBenedetti DB, Fehnel SE. Development and psychometric evaluation of the Endometriosis Treatment Satisfaction Questionnaire. Quality of Life Research. 2010;19: 899–905. doi:10.1007/s11136-010-9640-6

47. Fauconnier A, Staraci S, Daraï E, Descamps P, Nisolle M, Panel P, et al. A self-administered questionnaire to measure the painful symptoms of endometriosis: Results of a modified DELPHI

survey of patients and physicians. J Gynecol Obstet Hum Reprod. 2018;47: 69–79. doi:10.1016/j.jogoh.2017.11.003

48. Puchar A, Panel P, Oppenheimer A, Du Cheyron J, Fritel X, Fauconnier A. The ENDOPAIN 4D Questionnaire: A New Validated Tool for Assessing Pain in Endometriosis. J Clin Med. 2021;10: 3216. doi:10.3390/jcm10153216

49. Ahmadpour P, Jahangiry L, Bani S, Iravani M, Mirghafourvand M. Validation of the Iranian version of the ENDOPAIN-4D questionnaire for measurement of painful symptoms of endometriosis. J Obstet Gynaecol (Lahore). 2022;42. doi:10.1080/01443615.2022.2049726

50. As-Sanie S, Laufer MR, Missmer SA, Murji A, Vincent K, Eichner S, et al. Development of a visual, patient-reported tool for assessing the multi-dimensional burden of endometriosis. Curr Med Res Opin. 2021;37: 1443–1449. doi:10.1080/03007995.2021.1929896

51. Deal LS, Williams VSL, Fehnel SE. Development of an Electronic Daily Uterine Fibroid Symptom Diary. The Patient: Patient-Centered Outcomes Research. 2011;4: 31–44. doi:10.2165/11537290-000000000-00000

52. Li L, Huangfu L, Chai H, He W, Song H, Zou X, et al. Development of a functional and emotional measure of dysmenorrhea (FEMD) in Chinese university women. Health Care Women Int. 2012;33: 97–108. doi:10.1080/07399332.2011.603863

53. Yamada K, Adachi T, Kubota Y, Takeda T, Iseki M. Developing a Japanese version of the Injustice Experience Questionnaire-chronic and the contribution of perceived injustice to severity of menstrual pain: a web-based cross-sectional study. Biopsychosoc Med. 2019;13: 17. doi:10.1186/s13030-019-0158-z

54. Mansfield PK, Voda A, Allison G. Validating a pencil-and-paper measure of perimenopausal menstrual blood loss. Women's Health Issues. 2004;14: 242–247. doi:10.1016/j.whi.2004.07.005

55. Olliges E, Bobinger A, Weber A, Hoffmann V, Schmitz T, Popovici RM, et al. The Physical, Psychological, and Social Day-to-Day Experience of Women Living With Endometriosis Compared to Healthy Age-Matched Controls—A Mixed-Methods Study. Front Glob Womens Health. 2021;2: 767114. doi:10.3389/fgwh.2021.767114

56. Bushnell DM, Martin ML, Moore KA, Richter HE, Rubin A, Patrick DL. Menorrhagia Impact Questionnaire: assessing the influence of heavy menstrual bleeding on quality of life. Curr Med Res Opin. 2010;26: 2745–55. doi:10.1185/03007995.2010.532200

57. Shaw RW, Brickley MR, Evans L, Edwards MJ. Perceptions of women on the impact of menorrhagia on their health using multi-attribute utility assessment. BJOG. 1998;105: 1155–1159. doi:10.1111/j.1471-0528.1998.tb09968.x

58. Habiba M, Julian S, Taub N, Clark M, Rashid A, Baker R, et al. Limited role of multi-attribute utility scale and SF-36 in predicting management outcome of heavy menstrual bleeding. European Journal of Obstetrics & Gynecology and Reproductive Biology. 2010;148: 81–85. doi:10.1016/j.ejogrb.2009.09.021

59. Pattison H, Daniels J, Kai J, Gupta J. The measurement properties of the menorrhagia multi-attribute quality-of-life scale: a psychometric analysis. BJOG. 2011;118: 1528–1531. doi:10.1111/j.1471-0528.2011.03057.x

60. Brooks-Gunn J, Ruble DN. The menstrual attitude questionnaire. Psychosom Med. 1980;42: 503–12. doi:10.1097/00006842-198009000-00005

61. Bramwell RS, Biswas EL, Anderson C. Using the Menstrual Attitude Questionnaire with a British and an Indian sample. J Reprod Infant Psychol. 2002;20: 159–170. doi:10.1080/026468302760270818

62. Firat MZ, Kulakaç Ö, Öncel S, Akcan A. Menstrual Attitude Questionnaire: confirmatory and exploratory factor analysis with Turkish samples. J Adv Nurs. 2009;65: 652–662. doi:10.1111/j.1365-2648.2008.04919.x

63. Bargiota S, Bonotis K, Garyfallos G, Messinis I, Angelopoulos N. The Psychometric Properties of Menstrual Attitudes Questionnaire: A validity Study in Greek Women. Int J Innov Res Sci Eng Technol. 2016;5: 1754–1765. doi:10.15680/IJIRSET.2016.0502109

64. Kawata R, Endo M, Rai SK, Ohashi K. Development of a scale to evaluate negative menstrual attitudes among Nepalese women. Reprod Health. 2022;19: 120. doi:10.1186/s12978-022-01426-6

65. Rezende GP, Brito LGO, Gomes DAY, Souza LM de, Polo S, Benetti-Pinto CL. Assessing a cut-off point for the diagnosis of abnormal uterine bleeding using the Menstrual Bleeding Questionnaire (MBQ): a validation and cultural translation study with Brazilian women. Sao Paulo Med J. 2023;142: e2022539. doi:10.1590/1516-3180.2022.0539.R2.100423

66. Matteson KA, Scott DM, Raker CA, Clark MA. The menstrual bleeding questionnaire: development and validation of a comprehensive patient-reported outcome instrument for heavy menstrual bleeding. BJOG. 2015;122: 681–9. doi:10.1111/1471-0528.13273

67. Pike M, Chopek A, NL Y, Usuba K, MJ B, McLaughlin R, et al. Quality of life in adolescents with heavy menstrual bleeding: Validation of the Adolescent Menstrual Bleeding Questionnaire (aMBQ). Res Pract Thromb Haemost. 2021;5: e12615. doi:10.1002/rth2.12615

68. Rodpetch T, Manonai J, Angchaisuksiri P, Boonyawat K. A quality-of-life questionnaire for heavy menstrual bleeding in Thai women receiving oral antithrombotics: Assessment of the translated Menstrual Bleeding Questionnaire. Res Pract Thromb Haemost. 2021;5: e12617. doi:10.1002/rth2.12617

69. Toxqui L, Pérez-Granados AM, Blanco-Rojo R, Wright I, Vaquero MP. A simple and feasible questionnaire to estimate menstrual blood loss: relationship with hematological and gynecological parameters in young women. BMC Womens Health. 2014;14: 71. doi:10.1186/1472-6874-14-71

70. Chimbira TH, Anderson AB, Turnbull A c. Relation between measured menstrual blood loss and patient's subjective assessment of loss, duration of bleeding, number of sanitary towels used,

uterine weight and endometrial surface area. Br J Obstet Gynaecol. 1980;87: 603–9. doi:10.1111/j.1471-0528.1980.tb05013.x

71. Gleeson N, Devitt M, Buggy F, Bonnar J. Menstrual Blood Loss Measurement with Gynaeseal. Australian and New Zealand Journal of Obstetrics and Gynaecology. 1993;33: 79–80. doi:10.1111/j.1479-828X.1993.tb02061.x

72. Gannon MJ, Day P, Hammadieh N, Johnson N. A new method for measuring menstrual blood loss and its use in screening women before endometrial ablation. BJOG. 1996;103: 1029–1033. doi:10.1111/j.1471-0528.1996.tb09556.x

73. Fraser IS, Warner P, Marantos PA. Estimating menstrual blood loss in women with normal and excessive menstrual fluid volume. Obstetrics and gynecology. 2001;98: 806–14. doi:10.1016/s0029-7844(01)01581-2

74. Gudmundsdottir BR, Hjaltalin EF, Bragadottir G, Hauksson A, Geirsson RT, Onundarson PT. Quantification of menstrual flow by weighing protective pads in women with normal, decreased or increased menstruation. Acta Obstet Gynecol Scand. 2009;88: 275–9. doi:10.1080/00016340802673162

75. Moos RH. The development of a menstrual distress questionnaire. Psychosom Med. 1968;30: 853–67. doi:10.1097/00006842-196811000-00006

76. Lee AM, So-Kum Tang C, Chong C. A culturally sensitive study of premenstrual and menstrual symptoms among Chinese women. Journal of Psychosomatic Obstetrics & Gynecology. 2009;30: 105–114. doi:10.1080/01674820902789241

77. Vannuccini S, Rossi E, Cassioli E, Cirone D, Castellini G, Ricca V, et al. Menstrual Distress Questionnaire (MEDI-Q): a new tool to assess menstruation-related distress. Reprod Biomed Online. 2021;43: 1107–1116. doi:10.1016/j.rbmo.2021.08.029

78. Cassioli E, Rossi E, Melani G, Faldi M, Rellini AH, Wyatt RB, et al. The menstrual distress questionnaire (MEDI-Q): reliability and validity of the English version. Gynecological Endocrinology. 2023;39. doi:10.1080/09513590.2023.2227275

79. Shin H, Park Y-J, Cho I. Development and psychometric validation of the Menstrual Health Instrument (MHI) for adolescents in Korea. Health Care Women Int. 2018;39: 1090–1109. doi:10.1080/07399332.2017.1423487

80. Darabi F, Yaseri M, Rohban A, Khalajabadi-Farahani F. Development and Psychometric Properties of Menstrual Health Seeking Behaviors Questionnaire (MHSBQ-42) in Female Adolescents. J Reprod Infertil. 2018;19: 229–236. Available: http://www.ncbi.nlm.nih.gov/pubmed/30746338

81. Ramaiya A, Sood S. What are the psychometric properties of a menstrual hygiene management scale: a community-based cross-sectional study. BMC Public Health. 2020;20: 525. doi:10.1186/s12889-020-08627-3

82. Caruso BA, Portela G, McManus S, Clasen T. Assessing Women's Menstruation Concerns and Experiences in Rural India: Development and Validation of a Menstrual Insecurity Measure. Int J Environ Res Public Health. 2020;17: 3468. doi:10.3390/ijerph17103468

83. Aubeeluck A, Maguire M. The Menstrual Joy Questionnaire Items Alone Can Positively Prime Reporting of Menstrual Attitudes and Symptoms. Psychol Women Q. 2002;26: 160–162. doi:10.1111/1471-6402.00054

84. Hennegan J, Nansubuga A, Akullo A, Smith C, Schwab KJ. The Menstrual Practices Questionnaire (MPQ): development, elaboration, and implications for future research. Glob Health Action. 2020;13: 1829402. doi:10.1080/16549716.2020.1829402

85. Heath AL, Skeaff CM, Gibson RS. Validation of a questionnaire method for estimating extent of menstrual blood loss in young adult women. J Trace Elem Med Biol. 1999;12: 231–5. doi:10.1016/S0946-672X(99)80063-7

86. Roberts T-A. Female Trouble: The Menstrual Self-Evaluation Scale and Women's Self-Objectification. Psychol Women Q. 2004;28: 22–26. doi:10.1111/j.1471-6402.2004.00119.x

87. Garg S, Marimuthu Y, Bhatnagar N, Singh MmC, Borle A, Basu S, et al. Development and validation of a menstruation-related activity restriction questionnaire among adolescent girls in urban resettlement colonies of Delhi. Indian Journal of Community Medicine. 2021;46: 57. doi:10.4103/ijcm.IJCM_183_20

88. Trego LL. Development of the Military Women's Attitudes Toward Menstrual Suppression Scale: From Construct Definition to Pilot Testing. J Nurs Meas. 2009;17: 45–72. doi:10.1891/1061-3749.17.1.45

89. Farquhar CM, Roberts H, Okonkwo QL, Stewart AW. A pilot survey of the impact of menstrual cycles on adolescent health. Aust N Z J Obstet Gynaecol. 2009;49: 531–6. doi:10.1111/j.1479-828X.2009.01062.x

90. de Arruda GT, Driusso P, Rodrigues JC, de Godoy AG, Avila MA. Numerical rating scale for dysmenorrhea-related pain: a clinimetric study. Gynecological Endocrinology. 2022;38. doi:10.1080/09513590.2022.2099831

91. Pokrzywinski RM, Soliman AM, Snabes MC, Chen J, Taylor HS, Coyne KS. Responsiveness and thresholds for clinically meaningful changes in worst pain numerical rating scale for dysmenorrhea and nonmenstrual pelvic pain in women with moderate to severe endometriosis. Fertil Steril. 2021;115: 423–430. doi:10.1016/j.fertnstert.2020.07.013

92. Rodrigues JC, Avila MA, dos Reis FJJ, Carlessi RM, Godoy AG, Arruda GT, et al. 'Painting my pain': the use of pain drawings to assess multisite pain in women with primary dysmenorrhea. BMC Womens Health. 2022;22. doi:10.1186/s12905-022-01945-1

93. Parker MA, Kent AL, Sneddon A, Wang J, Shadbolt B. The Menstrual Disorder of Teenagers (MDOT) Study No. 2: Period ImPact and Pain Assessment (PIPPA) Tool Validation in a Large Population-Based Cross-Sectional Study of Australian Teenagers. J Pediatr Adolesc Gynecol. 2022;35: 30–38. doi:10.1016/j.jpag.2021.06.003

94. Lancastle D, Kopp Kallner H, Hale G, Wood B, Ashcroft L, Driscoll H. Development of a brief menstrual quality of life measure for women with heavy menstrual bleeding. BMC Womens Health. 2023;23. doi:10.1186/s12905-023-02235-0

95. Higham JM, O'Brien PM, Shaw RW. Assessment of menstrual blood loss using a pictorial chart. Br J Obstet Gynaecol. 1990;97: 734–9. doi:10.1111/j.1471-0528.1990.tb16249.x

96. Janssen CA, Scholten PC, Heintz AP. A simple visual assessment technique to discriminate between menorrhagia and normal menstrual blood loss. Obstetrics and gynecology. 1995;85: 977–82. doi:10.1016/0029-7844(95)00062-V

97. Barr F, Brabin L, Agbaje O. A pictorial chart for managing common menstrual disorders in Nigerian adolescents. Int J Gynaecol Obstet. 1999;66: 51–3. doi:10.1016/s0020-7292(99)00025-9

98. Reid PC, Coker A, Coltart R. Assessment of menstrual blood loss using a pictorial chart: a validation study. BJOG. 2000;107: 320–322. doi:10.1111/j.1471-0528.2000.tb13225.x

99. Wyatt KM, Dimmock PW, Walker TJ, O'Brien PMS. Determination of total menstrual blood loss. Fertil Steril. 2001;76: 125–131. doi:10.1016/S0015-0282(01)01847-7

100. Sanchez J, Andrabi S, Bercaw JL, Dietrich JE. Quantifying the PBAC in a Pediatric and Adolescent Gynecology Population. Pediatr Hematol Oncol. 2012;29: 479–484. doi:10.3109/08880018.2012.699165

101. Larsen L, Coyne K, Chwalisz K. Validation of the menstrual pictogram in women with leiomyomata associated with heavy menstrual bleeding. Reprod Sci. 2013;20: 680–7. doi:10.1177/1933719112463252

102. Magnay JL, Nevatte TM, Seitz C, O'Brien S. A new menstrual pictogram for use with feminine products that contain superabsorbent polymers. Fertil Steril. 2013;100: 1715-21.e1–4. doi:10.1016/j.fertnstert.2013.08.028

103. Magnay JL, Nevatte TM, O'Brien S, Gerlinger C, Seitz C. Validation of a new menstrual pictogram (superabsorbent polymer-c version) for use with ultraslim towels that contain superabsorbent polymers. Fertil Steril. 2014;101: 515–22. doi:10.1016/j.fertnstert.2013.10.051

104. Hald K, Lieng M. Assessment of Periodic Blood Loss: Interindividual and Intraindividual Variations of Pictorial Blood Loss Assessment Chart Registrations. J Minim Invasive Gynecol. 2014;21: 662–668. doi:10.1016/j.jmig.2014.01.015

105. Haberland C, Filonenko A, Seitz C, Börner M, Gerlinger C, Doll H, et al. Validation of a menstrual pictogram and a daily bleeding diary for assessment of uterine fibroid treatment efficacy in clinical studies. J Patient Rep Outcomes. 2020;4: 97. doi:10.1186/s41687-020-00263-0

106. Weller A, Weller L. Assessment of menstrual regularity and irregularity using self-reports and objective criteria. Journal of Psychosomatic Obstetrics & Gynecology. 1998;19: 111–116. doi:10.3109/01674829809048504

107. Schumacher U, Schumacher J, Mellinger U, Gerlinger C, Wienke A, Endrikat J. Estimation of menstrual blood loss volume based on menstrual diary and laboratory data. BMC Womens Health. 2012;12: 24. doi:10.1186/1472-6874-12-24

108. Wegienka G, Baird DD. A comparison of recalled date of last menstrual period with prospectively recorded dates. J Womens Health (Larchmt). 2005;14: 248–52. doi:10.1089/jwh.2005.14.248

109. Jukic AMZ, Weinberg CR, Baird DD, Hornsby PP, Wilcox AJ. Measuring menstrual discomfort: a comparison of interview and diary data. Epidemiology. 2008;19: 846–50. doi:10.1097/EDE.0b013e318187ac9e

110. Bachand AM, Cragin LA, Reif JS. Reliability of Retrospectively Assessed Categorical Menstrual Cycle Length Data. Ann Epidemiol. 2009;19: 501–503. doi:10.1016/j.annepidem.2009.03.015

111. Jukic AMZ, Weinberg CR, Wilcox AJ, McConnaughey DR, Hornsby P, Baird DD. Accuracy of Reporting of Menstrual Cycle Length. Am J Epidemiol. 2007;167: 25–33. doi:10.1093/aje/kwm265

112. Small CM, Manatunga AK, Marcus M. Validity of Self-Reported Menstrual Cycle Length. Ann Epidemiol. 2007;17: 163–170. doi:10.1016/j.annepidem.2006.05.005

113. Calaf J, Cancelo MJ, Andeyro M, Jiménez JM, Perelló J, Correa M, et al. Development and Psychometric Validation of a Screening Questionnaire to Detect Excessive Menstrual Blood Loss That Interferes in Quality of Life: The SAMANTA Questionnaire. J Womens Health (Larchmt). 2020;29: 1021–1031. doi:10.1089/jwh.2018.7446

114. Perelló-Capo J, Rius-Tarruella J, Andeyro-García M, Calaf-Alsina J. Sensitivity to Change of the SAMANTA Questionnaire, a Heavy Menstrual Bleeding Diagnostic Tool, after 1 Year of Hormonal Treatment. J Womens Health. 2023;32. doi:10.1089/jwh.2022.0155

115. Pérez-Campos E, Dueñas JL, de la Viuda E, Gómez MÁ, Lertxundi R, Sánchez-Borrego R, et al. Development and validation of the SEC-QOL questionnaire in women using contraceptive methods. Value Health. 2011;14: 892–9. doi:10.1016/j.jval.2011.08.1729

116. Kantarovich D, Dillane KE, Garrison EF, Oladosu FA, Schroer MS, Roth GE, et al. Development and validation of a real-time method characterizing spontaneous pain in women with dysmenorrhea. Journal of Obstetrics and Gynaecology Research. 2021;47: 1472–1480. doi:10.1111/jog.14663

117. Spies JB, Coyne K, Guaou Guaou N, Boyle D, Skyrnarz-Murphy K, Gonzalves SM. The UFS-QOL, a new disease-specific symptom and health-related quality of life questionnaire for leiomyomata. Obstetrics and gynecology. 2002;99: 290–300. doi:10.1016/s0029-7844(01)01702-1

118. Harding G, Coyne KS, Thompson CL, Spies JB. The responsiveness of the uterine fibroid symptom and health-related quality of life questionnaire (UFS-QOL). Health Qual Life Outcomes. 2008;6: 99. doi:10.1186/1477-7525-6-99

119. Silva R, Gomes M, Castro R, Bonduki C, Girão M. Uterine Fibroid Symptom - Quality of Life questionnaire translation and validation into Brazilian Portuguese. Revista Brasileira de Ginecologia e Obstetrícia / RBGO Gynecology and Obstetrics. 2016;38: 518–523. doi:10.1055/s-0036-1593833

120. Oliveira Brito LG, Malzone-Lott DA, Sandoval Fagundes MF, Magnani PS, Fernandes Arouca MA, Poli-Neto OB, et al. Translation and validation of the Uterine Fibroid Symptom and Quality of Life (UFS-QOL) questionnaire for the Brazilian Portuguese language. Sao Paulo Medical Journal. 2017;135: 107–115. doi:10.1590/1516-3180.2016.0223281016

121. Coyne KS, Soliman AM, Margolis MK, Thompson CL, Chwalisz K. Validation of the 4 week recall version of the Uterine Fibroid Symptom and Health-related Quality of Life (UFS-QOL) Questionnaire. Curr Med Res Opin. 2017;33: 193–200. doi:10.1080/03007995.2016.1248382

122. Coyne KS, Harrington A, Currie BM, Chen J, Gillard P, Spies JB. Psychometric validation of the 1-month recall Uterine Fibroid Symptom and Health-Related Quality of Life questionnaire (UFS-QOL). J Patient Rep Outcomes. 2019;3: 57. doi:10.1186/s41687-019-0146-x

123. Yeung S, Kwok JW, Law S, Chung JP, Chan SS. Uterine Fibroid Symptom and Health-related Quality of Life Questionnaire: a Chinese translation and validation study. Hong Kong Medical Journal. 2019;25: 453–459. doi:10.12809/hkmj198064

124. Calaf J, Palacios S, Cristóbal I, Cañete ML, Monleón J, Fernández J, et al. Validation of the Spanish version of the Uterine Fibroid Symptom and Quality of Life (UFS-QoL) questionnaire in women with uterine myomatosis. Med Clin (Barc). 2020;154: 207–213. doi:10.1016/j.medcli.2019.05.027

125. Keizer AL, van Kesteren PJM, Terwee C, de Lange ME, Hehenkamp WJK, Kok HS. Uterine Fibroid Symptom and Quality of Life questionnaire (UFS-QOL NL) in the Dutch population: a validation study. BMJ Open. 2021;11: e052664. doi:10.1136/bmjopen-2021-052664

126. Gerlinger C, Schumacher U, Wentzeck R, Uhl-Hochgräber K, Solomayer EF, Schmitz H, et al. How can we measure endometriosis-associated pelvic pain? J Endometr. 2012;4: 109–116. doi:10.5301/JE.2012.9725

127. Gerlinger C, Schumacher U, Faustmann T, Colligs A, Schmitz H, Seitz C. Defining a minimal clinically important difference for endometriosis-associated pelvic pain measured on a visual analog scale: analyses of two placebo-controlled, randomized trials. Health Qual Life Outcomes. 2010;8: 138. doi:10.1186/1477-7525-8-138

128. Perelló J, Pujol P, Pérez M, Artés M, Calaf J. Heavy Menstrual Bleeding-Visual Analog Scale, an Easy-to-Use Tool for Excessive Menstrual Blood Loss That Interferes with Quality-of-Life Screening in Clinical Practice. Womens Health Rep (New Rochelle). 2022;3: 483–490. doi:10.1089/whr.2021.0139

129. de Arruda GT, de Melo Mantovan SG, Da Roza T, Silva BI da, Tonon da Luz SC, Avila MA. WHODAS measurement properties for women with dysmenorrhea. Health Qual Life Outcomes. 2023;21. doi:10.1186/s12955-023-02140-y

130. Teherán A, Pineros LG, Pulido F, Mejía Guatibonza MC. WaLIDD score, a new tool to diagnose dysmenorrhea and predict medical leave in university students. Int J Womens Health. 2018;10: 35–45. doi:10.2147/IJWH.S143510

131. Yu S-C, Hsu H-P, Guo J-L, Chen S-F, Huang S-H, Chen Y-C, et al. Exploration of the experiences of working stressors and coping strategies associated with menstrual symptoms among nurses with

shifting schedules: a Q methodology investigation. BMC Nurs. 2021;20: 238. doi:10.1186/s12912-021-00759-0

132. Vitonis AF, Vincent K, Rahmioglu N, Fassbender A, Buck Louis GM, Hummelshoj L, et al. World Endometriosis Research Foundation Endometriosis Phenome and biobanking harmonization project: II. Clinical and covariate phenotype data collection in endometriosis research. Fertil Steril. 2014;102: 1223–1232. doi:10.1016/j.fertnstert.2014.07.1244

133. Dimentberg E, Cardaillac C, Richard E, Plante A-S, Maheux-Lacroix S. Translation and Cultural Validation of the WERF EPHect Endometriosis Patient Questionnaire into Canadian French. Journal of Obstetrics and Gynaecology Canada. 2021;43: 817–821. doi:10.1016/j.jogc.2021.03.019

134. Melin A, Tornberg ÅB, Skouby S, Faber J, Ritz C, Sjödin A, et al. The LEAF questionnaire: a screening tool for the identification of female athletes at risk for the female athlete triad. Br J Sports Med. 2014;48: 540–545. doi:10.1136/bjsports-2013-093240

135. de Maria UP, Juzwiak CR. Cultural adaptation and validation of the Low Energy Availability in Females Questionnaire (LEAF-Q). Revista Brasileira de Medicina do Esporte. 2021;27: 184–188. doi:10.1590/1517-869220212702223889

136. Sutthibut K, Itharat A, Singchungchai P, Wanichsetakul P, Pipatrattanaseree W, Ooraikul B, et al. Development of an Assessment Tool of Menstrual-Cycle-Related Signs and Symptoms Based on Thai Traditional Medicine Principles for Evaluation of Women's Health. Shang H, editor. Evidence-Based Complementary and Alternative Medicine. 2021;2021: 1–16. doi:10.1155/2021/9977773

137. Chesney MA, Tasto DL. The development of the menstrual symptom questionnaire. Behaviour research and therapy. 1975;13: 237–44. doi:10.1016/0005-7967(75)90028-5

138. Nelson RO, Sigmon S, Amodei N, Jarrett RB. The menstrual symptom questionnaire: The validity of the distinction between spasmodic and congestive dysmenorrhea. Behaviour Research and Therapy. 1984;22: 611–614. doi:10.1016/0005-7967(84)90123-2

139. Negriff S, Dorn LD, Hillman JB, Huang B. The measurement of menstrual symptoms: factor structure of the menstrual symptom questionnaire in adolescent girls. J Health Psychol. 2009;14: 899–908. doi:10.1177/1359105309340995

140. Im E-O. The Midlife Women's Symptom Index (MSI). Health Care Women Int. 2006;27: 268–287. doi:10.1080/07399330500506600

141. Mathias SD, Colwell HH, LoCoco JM, Karvois DL, Pritchard ML, Friedman AJ. ORTHO birth control satisfaction assessment tool: assessing sensitivity to change and predictors of satisfaction. Contraception. 2006;74: 303–308. doi:10.1016/j.contraception.2006.03.033

142. Colwell HH, Mathias SD, Cimms TA, Rothman M, Friedman AJ, Patrick DL. The ORTHO BC-SAT – a satisfaction questionnaire for women using hormonal contraceptives. Quality of Life Research. 2006;15: 1621–1631. doi:10.1007/s11136-006-0026-8

143. Mahalingaiah S, Cosenza C, Cheng JJ, Rodriguez E, Aschengrau A. Cognitive testing of a survey instrument for self-assessed menstrual cycle characteristics and androgen excess. Fertil Res Pract. 2020;6: 19. doi:10.1186/s40738-020-00088-x

144. Saei Ghare Naz M, Ozgoli G, Ahmadi F, Alavi Majd H, Aflatounian A, Ramezani Tehrani F. Adolescents' polycystic ovary syndrome health-related quality of life questionnaire (APQ-20): development and psychometric properties. Eur J Pediatr. 2023;182. doi:10.1007/s00431-023-04875-8

145. Cronin L, Guyatt G, Griffith L, Wong E, Azziz R, Futterweit W, et al. Development of a health-related quality-of-life questionnaire (PCOSQ) for women with polycystic ovary syndrome (PCOS). J Clin Endocrinol Metab. 1998;83: 1976–87. doi:10.1210/jcem.83.6.4990

146. Jones GL, Benes K, Clark TL, Denham R, Holder MG, Haynes TJ, et al. The Polycystic Ovary Syndrome Health-Related Quality of Life Questionnaire (PCOSQ): a validation. Hum Reprod. 2004;19: 371–7. doi:10.1093/humrep/deh048

147. Guyatt G, Weaver B, Cronin L, Dooley JA, Azziz R. Health-related quality of life in women with polycystic ovary syndrome, a self-administered questionnaire, was validated. J Clin Epidemiol. 2004;57: 1279–1287. doi:10.1016/j.jclinepi.2003.10.018

148. Jedel E, Kowalski J, Stener-Victorin E. Assessment of health-related quality of life: Swedish version of polycystic ovary syndrome questionnaire. Acta Obstet Gynecol Scand. 2008;87: 1329–35. doi:10.1080/00016340802444762

149. Bazarganipour F, Ziaei S, Montazeri A, Faghihzadeh S, Frozanfard F. Psychometric properties of the Iranian version of modified polycystic ovary syndrome health-related quality-of-life questionnaire. Human Reproduction. 2012;27: 2729–2736. doi:10.1093/humrep/des199

150. Bazarganipour F, Ziaei S, Montazeri A, Foroozanfard F, Faghihzadeh S. Iranian version of modified polycystic ovary syndrome health-related quality of Life questionnaire: Discriminant and convergent validity. Iran J Reprod Med. 2013;11: 753–60. Available: http://www.ncbi.nlm.nih.gov/pubmed/24639816

151. Chung J, Kwan A, Kwok J, Chan S. Health-related quality-of-life questionnaire for women with polycystic ovary syndrome: a Chinese translation and validation study. BJOG. 2016;123: 1638–45. doi:10.1111/1471-0528.14217

152. Nikpour M, Tirgar A, Ghaffari F, Ebadi A, Sharif Nia H, Nasiri-Amiri F. Development and psychometric evaluation of the women shift workers' reproductive health questionnaire: a sequential exploratory mixed-method study. Reprod Health. 2020;17: 147. doi:10.1186/s12978-020-00994-9

153. Morse JM, Kieren D, Bottorff J. The adolescent menstrual attitude questionnaire, part I: Scale construction. Health Care Women Int. 1993;14: 39–62. doi:10.1080/07399339309516025

154. Morse JM, Kieren D. The adolescent menstrual attitude questionnaire, part II: Normative scores. Health Care Women Int. 1993;14: 63–76. doi:10.1080/07399339309516026

155. Whitham HK, MacLehose RF, Harlow BL, Wellons MF, Schreiner PJ. Assessing the utility of methods for menopausal transition classification in a population-based cohort: The CARDIA Study. Maturitas. 2013;75: 289–293. doi:10.1016/j.maturitas.2013.04.015

156. Pedersen SD, Brar S, Faris P, Corenblum B. Polycystic ovary syndrome: validated questionnaire for use in diagnosis. Can Fam Physician. 2007;53: 1042–7, 1041. Available: http://www.ncbi.nlm.nih.gov/pubmed/17872783

157. Chapron C, Lafay-Pillet M-C, Santulli P, Bourdon M, Maignien C, Gaudet-Chardonnet A, et al. A new validated screening method for endometriosis diagnosis based on patient questionnaires. EClinicalMedicine. 2022;44: 101263. doi:10.1016/j.eclinm.2021.101263

158. van Barneveld E, Lim A, van Hanegem N, van Osch F, Vork L, Kruimel J, et al. Real-time Symptom Assessment in Patients With Endometriosis: Psychometric Evaluation of an Electronic Patient-Reported Outcome Measure, Based on the Experience Sampling Method. JMIR Form Res. 2023;7. doi:10.2196/29480

159. Mathias SD, Bussel JB, George JN, McMillan R, Okano GJ, Nichol JL. A disease-specific measure of health-related quality of life in adults with chronic immune thrombocytopenic purpura: psychometric testing in an open-label clinical trial. Clin Ther. 2007;29: 950–962. doi:10.1016/j.clinthera.2007.05.005

160. DiBenedetti DB, Soliman AM, Ervin C, Evans E, Coddington CC, Agarwal SK, et al. Development of the Painful Periods Screening Tool for endometriosis. Postgrad Med. 2018;130: 694–702. doi:10.1080/00325481.2018.1526623

161. Nasiri-Amiri F, Ramezani Tehrani F, Simbar M, Montazeri A, Mohammadpour RA. Health-related quality of life questionnaire for polycystic ovary syndrome (PCOSQ-50): development and psychometric properties. Quality of Life Research. 2016;25: 1791–1801. doi:10.1007/s11136-016-1232-7

162. Stevanovic D, Bozic-Antic I, Stanojlovic O, Vojnovic Milutinovic D, Bjekic-Macut J, Jancic J, et al. Health-related quality of life questionnaire for polycystic ovary syndrome (PCOSQ-50): a psychometric study with the Serbian version. Women Health. 2019;59: 1015–1025. doi:10.1080/03630242.2019.1587664

163. Hunter EC, Murray SM, Sultana F, Alam MU, Sarker S, Rahman M, et al. Development and validation of the Self-Efficacy in Addressing Menstrual Needs Scale (SAMNS-26) in Bangladeshi schools: A measure of girls' menstrual care confidence. PLoS One. 2022;17. doi:10.1371/journal.pone.0275736

164. Rizwana R, Ashraf K. The construction and validation of the Stellenbosch Endometriosis Quality of life measure (SEQOL). Health Care Women Int. 2018;39: 1123–1139. doi:10.1080/07399332.2018.1455684

165. Hackethal A, Luck C, von Hobe A-K, Eskef K, Oehmke F, Konrad L. A structured questionnaire improves preoperative assessment of endometriosis patients: a retrospective analysis and prospective trial. Arch Gynecol Obstet. 2011;284: 1179–1188. doi:10.1007/s00404-010-1819-0

166. Alexander MS, Biering-Sørensen F, Elliott S, Kreuter M, Sønksen J. International Spinal Cord Injury Female Sexual and Reproductive Function Basic Data Set. Spinal Cord. 2011;49: 787–790. doi:10.1038/sc.2011.7

167. Pokrzywinski RM, Soliman AM, Chen J, Snabes MC, Agarwal SK, Coddington C, et al. Psychometric assessment of the health-related productivity questionnaire (HRPQ) among women with endometriosis. Expert Rev Pharmacoecon Outcomes Res. 2020;20: 531–539. doi:10.1080/14737167.2019.1662301

168. Ruta DA, Garratt AM, Russell IT. Patient centred assessment of quality of life for patients with four common conditions. Qual Saf Health Care. 1999;8: 22–29. doi:10.1136/qshc.8.1.22

169. Schneider S, Broderick JE, Junghaenel DU, Schwartz JE, Stone AA. Temporal trends in symptom experience predict the accuracy of recall PROs. J Psychosom Res. 2013;75: 160–166. doi:10.1016/j.jpsychores.2013.06.006

170. Pokrzywinski R, Soliman AM, Surrey E, Snabes MC, Coyne KS. Psychometric assessment of the PROMIS Fatigue Short Form 6a in women with moderate-to-severe endometriosis-associated pain. J Patient Rep Outcomes. 2020;4: 86. doi:10.1186/s41687-020-00257-y

171. Ferreira-Valente A, Garcia IQ, Rosa AM, Pereira A, Pais-Ribeiro JL, Jensen MP. The Portuguese 35-item Survey of Pain Attitudes applied to Portuguese women with Endometriosis. Scand J Pain. 2019;19: 553–563. doi:10.1515/sjpain-2019-0004

172. Hunter M. The women's health questionnaire: A measure of mid-aged women's perceptions of their emotional and physical health. Psychol Health. 1992;7: 45–54. doi:10.1080/08870449208404294

173. Hunter M. The Women's Health Questionnaire (WHQ): The development, standardization and application of a measure of mid-aged women's emotional and physical health. Quality of Life Research. 2000;9: 733–738. doi:10.1023/A:1008973822876

174. Colantonio A, Harris JE, Tarek N. Reliability of a Health Questionnaire Among Women With Brain Injury. Journal of Neuroscience Nursing. 2011;43: 141–148. doi:10.1097/JNN.0b013e3182135b13
