# Supplementary material for: Measurement of changes to the menstrual cycle: A transdisciplinary systematic review evaluating measure quality and utility for clinical trials
Source: PLoS One. 2024 Jul 25;19(7):e0306491. doi: 10.1371/journal.pone.0306491 (PMC11271926; doi:10.1371/journal.pone.0306491)
Supplement: S3 Table — (PDF) [file pone.0306491.s003.pdf]

S3 Table. Characteristics of sub-scales, items, and general instruments.

Full Name of instrument	Available Languages	Available Electronically?*	BLEEDING				BLOOD			UTERINE PAIN	PERCEP-TIONS	Subscale Topic or Wording of Questions	Ref
			Duration	Volume	Frequency	Regularity	Color	Consistency	Smell				
**Instruments with subscales on menstrual changes (n=8)**													
Low Energy Availability in Females Questionnaire	Danish, English, Portuguese, Swedish	Yes	X	X	X	X						Menstrual function and use of contraceptives	[1,2]
Menstrual Cycle-Related Signs and Symptoms Questionnaire	Thai	No		X			X	X	X		X	Menstrual cycle-related signs and symptoms (section 1)	[3]
Menstrual Symptom Questionnaire	English	No								X		Spasmodic dysmenorrhea, also called menstrual discomfort or factor 1: abdominal pain in later versions of the too	[4–6]
Midlife Women's Symptom Index	English	No		X	X	X						Self-reported menopausal status (includes last menstrual cycle, menstrual regularity, and menstrual flow)	[7]
ORTHO Birth Control Satisfaction Assessment Tool	English	No	X	X	X					X	X	Menstrual impact and lifestyle impact	[8,9]
Ovulation and Menstruation Health Survey	English	Yes	X	X		X						Menstrual cycle questions	[10]
Polycystic Ovary Syndrome Quality of Life Scale	Chinese, English, Persian, Swedish	No				X				X		Menstrual problems (menstruation or menstrual)	[11–18]
Women Shift Workers Reproductive Health Questionnaire	Persian	No				X				X		Menstruation	[19]
**Instruments with items or questions on menstrual changes (n=13)**													
Adolescent Menstrual Attitude Questionnaire	English	No				X				X	X	[strongly disagree; disagree; don't care, are not sure, or do not know; agree with the statement; strongly]: "I worry a lot about my periods starting unexpectedly."; "I do not feel any different than usual when I menstruate."; "I feel okay when I get my period."; "When I have my period, I feel good."; "When I get my period, I feel sick."; "Cramps during my period are very painful."; "I feel ugly and gross when I have my period."; "When I am menstruating I feel the same."	[20,21]
CARDIA Women's Reproductive Health Questionnaire	English	Yes				X						"During the past 12 months, have your menstrual cycles been regular at least half the time (excluding times when you were on birth control pills, pregnant, or nursing)?: [no, yes, not sure]?"	[22]
Clinical Tool for Diagnosis of PCOS	English	No			X							"Please answer this question NOT INCLUDING any time spent pregnant, receiving birth control pills or injections, after menopause, or after having both ovaries or the uterus surgically removed: Between the ages of 16 and 40, about how long was your average menstrual cycle (time from first day of one period to the first day of the next period)? Select ONE only: [<25 d, 25-34 d, 35-60 d, more than 60 d , totally variable]"	[23]
Clinically Validated Scores for Endometriosis Diagnosis**	French	No								X		Visual analogue scale (range: 0-10): dysmenorrhea ≥6	[24]
Experience Sampling Method	Dutch	Yes								X		"I suffer from abdominal pain."; "I feel pain when I am standing/walking."	[25]
Immune Thrombocytopenic Purpura Patient Assessment Questionnaire (ITP-PAQ)	Multi-Site Study	No	X	X						X	X	"Thinking about your last period.... How bothered were you by heavier bleeding before having ITP?"; "How bothered were you by bleeding for more days than before having ITP?"; "How bothered were you by more pain before ITP? " [5-point Likert scale from "extremely" to "not at all"]	[26]
Painful Periods Screening Tool	English	No								X	X	"Do you often experience pelvic/abdominal or lower back pain before or during your periods that limits your activities or requires medication?"; Do you sometimes avoid sexual intercourse to avoid pain? [Yes, No, Not applicable, I am not sexually active]"	[27]
Polycystic Ovary Syndrome Questionnaire-50	Persian, Serbian	No									X	"In the past 4 weeks have you ever...Felt concerned about menstruation at long intervals?" [Y/N]	[28,29]
Self-Efficacy in Addressing Menstrual Needs Scale	Bengali	Yes	X							X	X	"How confident are you that you can count/keep track of your period days?"; "How confident are you that you can usually reduce your abdominal pain by a small amount?"; "How confident are you that you can usually reduce most of your abdominal pain?"; "How confident are you that you can usually reduce your abdominal pain completely?"	[30]
Stellenbosch Endometriosis Quality of Life Measure	English	No						X			X	"I was concerned about the clots in my period."; "I was worried that my period was not normal."; "I felt that my period drained me." [not applicable, not at all, a little bit, somewhat, quite a bit, very much]	[31]

Full Name of instrument	Available Languages	Available Electronically?*	BLEEDING				BLOOD			UTERINE PAIN	PERCEP-TIONS	Subscale Topic or Wording of Questions	Ref
			Duration	Volume	Frequency	Regularity	Color	Consistency	Smell				
Structured Endometriosis Questionnaire	English, German	Yes		X		X				X		"Do you have a regular menstrual cycle? [Y/N]"; "How long is your average period?"; "How long is your average bleeding?"; "When was the first day of your last period?"; "How do you estimate the intensity of the last menstrual bleeding?"; "Do you have pain in connection with you menstrual bleeding? [Y/N], If yes, when do you feel pain [previously, meanwhile, afterwards] How strong do you feel the pain on a scale from 0 to 10?"	[32]
The International Spinal Cord Injury Female Sexual and Reproductive Function	English	No	X	X	X							"How would you rate your current menstruation pattern? [Normal, Reduced/altered, Absent, Unknown, Not applicable]"	[33]
Uterine Fibroid Daily Bleeding Diary	Multi-Site Study	No		X								"Rate the severity of any vaginal bleeding in the past 24 hours. [No vaginal bleeding, Spotting, Mild, Moderate, Severe, Very severe]"	[34]
**General instruments validate in menstruating populations (n=5)**													
Health Related Productivity Questionnaire	Multi-Site Study	Yes									X	NA (General questions applied to menstruating population but no questions specific to menstruation)	[35]
Patient-Generated Index of Quality of Life	English	No									X	"We would like you to think of the most important areas of your life that are affected by your Menorrhagia. Please write up to FIVE areas in the boxes below."	[36]
Patient-Reported Outcomes Measurement Information System	English	Yes	X							X	X	NA (General questions applied to menstruating population but no questions specific to menstruation)	[37,38]
Survey of Pain Attitudes	Portuguese	Yes								X	X	NA (General questions applied to menstruating population but no questions specific to menstruation)	[39]
Women's Health Questionnaire	English	No		X						X		"Please indicate how you are feeling now, or how you have been feeling THE LAST FEW DAYS, by putting a tick in the correct box in the answer to each of the following items [Yes definitely, yes sometimes, no not much, no not at all]: 'I have heavy periods.'; 'My breasts feel tender or uncomfortable.'; 'I have abdominal cramps or discomfort.'"	[40–42]

*According to publications, "Yes" indicates either fully or partly electronic

**All tools or subscales were designed to be completed by patients or participants, except for the Clinically Validated Scores for Endometriosis Diagnosis

References

1. de Maria UP, Juzwiak CR. Cultural adaptation and validation of the Low Energy Availability in Females Questionnaire (LEAF-Q). Revista Brasileira de Medicina do Esporte. 2021;27: 184–188. doi:10.1590/1517-869220212702223889

2. Melin A, Tornberg ÅB, Skouby S, Faber J, Ritz C, Sjödin A, et al. The LEAF questionnaire: a screening tool for the identification of female athletes at risk for the female athlete triad. Br J Sports Med. 2014;48: 540–545. doi:10.1136/bjsports-2013-093240

3. Sutthibut K, Itharat A, Singchungchai P, Wanichsetakul P, Pipatrattanaseree W, Ooraikul B, et al. Development of an Assessment Tool of Menstrual-Cycle-Related Signs and Symptoms Based on Thai Traditional Medicine Principles for Evaluation of Women's Health. Shang H, editor. Evidence-Based Complementary and Alternative Medicine. 2021;2021: 1–16. doi:10.1155/2021/9977773

4. Chesney MA, Tasto DL. The development of the menstrual symptom questionnaire. Behaviour research and therapy. 1975;13: 237–44. doi:10.1016/0005-7967(75)90028-5

5. Negriff S, Dorn LD, Hillman JB, Huang B. The measurement of menstrual symptoms: factor structure of the menstrual symptom questionnaire in adolescent girls. J Health Psychol. 2009;14: 899–908. doi:10.1177/1359105309340995

6. Nelson RO, Sigmon S, Amodei N, Jarrett RB. The menstrual symptom questionnaire: The validity of the distinction between spasmodic and congestive dysmenorrhea. Behaviour Research and Therapy. 1984;22: 611–614. doi:10.1016/0005-7967(84)90123-2

7. Im E-O. The Midlife Women's Symptom Index (MSI). Health Care Women Int. 2006;27: 268–287. doi:10.1080/07399330500506600

8. Colwell HH, Mathias SD, Cimms TA, Rothman M, Friedman AJ, Patrick DL. The ORTHO BC-SAT – a satisfaction questionnaire for women using hormonal contraceptives. Quality of Life Research. 2006;15: 1621–1631. doi:10.1007/s11136-006-0026-8

9. Mathias SD, Colwell HH, LoCoco JM, Karvois DL, Pritchard ML, Friedman AJ. ORTHO birth control satisfaction assessment tool: assessing sensitivity to change and predictors of satisfaction. Contraception. 2006;74: 303–308. doi:10.1016/j.contraception.2006.03.033

10. Mahalingaiah S, Cosenza C, Cheng JJ, Rodriguez E, Aschengrau A. Cognitive testing of a survey instrument for self-assessed menstrual cycle characteristics and androgen excess. Fertil Res Pract. 2020;6: 19. doi:10.1186/s40738-020-00088-x

11. Bazarganipour F, Ziaei S, Montazeri A, Faghihzadeh S, Frozanfard F. Psychometric properties of the Iranian version of modified polycystic ovary syndrome health-related quality-of-life questionnaire. Human Reproduction. 2012;27: 2729–2736. doi:10.1093/humrep/des199

12. Bazarganipour F, Ziaei S, Montazeri A, Foroozanfard F, Faghihzadeh S. Iranian version of modified polycystic ovary syndrome health-related quality of Life questionnaire: Discriminant and convergent validity. Iran J Reprod Med. 2013;11: 753–60. Available: http://www.ncbi.nlm.nih.gov/pubmed/24639816

13. Chung J, Kwan A, Kwok J, Chan S. Health-related quality-of-life questionnaire for women with polycystic ovary syndrome: a Chinese translation and validation study. BJOG. 2016;123: 1638–45. doi:10.1111/1471-0528.14217

14. Cronin L, Guyatt G, Griffith L, Wong E, Azziz R, Futterweit W, et al. Development of a health-related quality-of-life questionnaire (PCOSQ) for women with polycystic ovary syndrome (PCOS). J Clin Endocrinol Metab. 1998;83: 1976–87. doi:10.1210/jcem.83.6.4990

15. Guyatt G, Weaver B, Cronin L, Dooley JA, Azziz R. Health-related quality of life in women with polycystic ovary syndrome, a self-administered questionnaire, was validated. J Clin Epidemiol. 2004;57: 1279–1287. doi:10.1016/j.jclinepi.2003.10.018

16. Jedel E, Kowalski J, Stener-Victorin E. Assessment of health-related quality of life: Swedish version of polycystic ovary syndrome questionnaire. Acta Obstet Gynecol Scand. 2008;87: 1329–35. doi:10.1080/00016340802444762

17. Jones GL, Benes K, Clark TL, Denham R, Holder MG, Haynes TJ, et al. The Polycystic Ovary Syndrome Health-Related Quality of Life Questionnaire (PCOSQ): a validation. Hum Reprod. 2004;19: 371–7. doi:10.1093/humrep/deh048

18. Saei Ghare Naz M, Ozgoli G, Ahmadi F, Alavi Majd H, Aflatounian A, Ramezani Tehrani F. Adolescents' polycystic ovary syndrome health-related quality of life questionnaire (APQ-20): development and psychometric properties. Eur J Pediatr. 2023;182. doi:10.1007/s00431-023-04875-8

19. Nikpour M, Tirgar A, Ghaffari F, Ebadi A, Sharif Nia H, Nasiri-Amiri F. Development and psychometric evaluation of the women shift workers' reproductive health questionnaire: a sequential exploratory mixed-method study. Reprod Health. 2020;17: 147. doi:10.1186/s12978-020-00994-9

20. Morse JM, Kieren D, Bottorff J. The adolescent menstrual attitude questionnaire, part I: Scale construction. Health Care Women Int. 1993;14: 39–62. doi:10.1080/07399339309516025

21. Morse JM, Kieren D. The adolescent menstrual attitude questionnaire, part II: Normative scores. Health Care Women Int. 1993;14: 63–76. doi:10.1080/07399339309516026

22. Whitham HK, MacLehose RF, Harlow BL, Wellons MF, Schreiner PJ. Assessing the utility of methods for menopausal transition classification in a population-based cohort: The CARDIA Study. Maturitas. 2013;75: 289–293. doi:10.1016/j.maturitas.2013.04.015

23. Pedersen SD, Brar S, Faris P, Corenblum B. Polycystic ovary syndrome: validated questionnaire for use in diagnosis. Can Fam Physician. 2007;53: 1042–7, 1041. Available: http://www.ncbi.nlm.nih.gov/pubmed/17872783

24. Chapron C, Lafay-Pillet M-C, Santulli P, Bourdon M, Maignien C, Gaudet-Chardonnet A, et al. A new validated screening method for endometriosis diagnosis based on patient questionnaires. EClinicalMedicine. 2022;44: 101263. doi:10.1016/j.eclinm.2021.101263

25. van Barneveld E, Lim A, van Hanegem N, van Osch F, Vork L, Kruimel J, et al. Real-time Symptom Assessment in Patients With Endometriosis: Psychometric Evaluation of an Electronic Patient-

Reported Outcome Measure, Based on the Experience Sampling Method. JMIR Form Res. 2023;7. doi:10.2196/29480

26. Mathias SD, Bussel JB, George JN, McMillan R, Okano GJ, Nichol JL. A disease-specific measure of health-related quality of life in adults with chronic immune thrombocytopenic purpura: psychometric testing in an open-label clinical trial. Clin Ther. 2007;29: 950–962. doi:10.1016/j.clinthera.2007.05.005

27. DiBenedetti DB, Soliman AM, Ervin C, Evans E, Coddington CC, Agarwal SK, et al. Development of the Painful Periods Screening Tool for endometriosis. Postgrad Med. 2018;130: 694–702. doi:10.1080/00325481.2018.1526623

28. Nasiri-Amiri F, Ramezani Tehrani F, Simbar M, Montazeri A, Mohammadpour RA. Health-related quality of life questionnaire for polycystic ovary syndrome (PCOSQ-50): development and psychometric properties. Quality of Life Research. 2016;25: 1791–1801. doi:10.1007/s11136-016-1232-7

29. Stevanovic D, Bozic-Antic I, Stanojlovic O, Vojnovic Milutinovic D, Bjekic-Macut J, Jancic J, et al. Health-related quality of life questionnaire for polycystic ovary syndrome (PCOSQ-50): a psychometric study with the Serbian version. Women Health. 2019;59: 1015–1025. doi:10.1080/03630242.2019.1587664

30. Hunter EC, Murray SM, Sultana F, Alam MU, Sarker S, Rahman M, et al. Development and validation of the Self-Efficacy in Addressing Menstrual Needs Scale (SAMNS-26) in Bangladeshi schools: A measure of girls' menstrual care confidence. PLoS One. 2022;17. doi:10.1371/journal.pone.0275736

31. Rizwana R, Ashraf K. The construction and validation of the Stellenbosch Endometriosis Quality of life measure (SEQOL). Health Care Women Int. 2018;39: 1123–1139. doi:10.1080/07399332.2018.1455684

32. Hackethal A, Luck C, von Hobe A-K, Eskef K, Oehmke F, Konrad L. A structured questionnaire improves preoperative assessment of endometriosis patients: a retrospective analysis and prospective trial. Arch Gynecol Obstet. 2011;284: 1179–1188. doi:10.1007/s00404-010-1819-0

33. Alexander MS, Biering-Sørensen F, Elliott S, Kreuter M, Sønksen J. International Spinal Cord Injury Female Sexual and Reproductive Function Basic Data Set. Spinal Cord. 2011;49: 787–790. doi:10.1038/sc.2011.7

34. Haberland C, Filonenko A, Seitz C, Börner M, Gerlinger C, Doll H, et al. Validation of a menstrual pictogram and a daily bleeding diary for assessment of uterine fibroid treatment efficacy in clinical studies. J Patient Rep Outcomes. 2020;4: 97. doi:10.1186/s41687-020-00263-0

35. Pokrzywinski RM, Soliman AM, Chen J, Snabes MC, Agarwal SK, Coddington C, et al. Psychometric assessment of the health-related productivity questionnaire (HRPQ) among women with endometriosis. Expert Rev Pharmacoecon Outcomes Res. 2020;20: 531–539. doi:10.1080/14737167.2019.1662301

36. Ruta DA, Garratt AM, Russell IT. Patient centred assessment of quality of life for patients with four common conditions. Qual Saf Health Care. 1999;8: 22–29. doi:10.1136/qshc.8.1.22

37. Pokrzywinski R, Soliman AM, Surrey E, Snabes MC, Coyne KS. Psychometric assessment of the PROMIS Fatigue Short Form 6a in women with moderate-to-severe endometriosis-associated pain. J Patient Rep Outcomes. 2020;4: 86. doi:10.1186/s41687-020-00257-y

38. Schneider S, Broderick JE, Junghaenel DU, Schwartz JE, Stone AA. Temporal trends in symptom experience predict the accuracy of recall PROs. J Psychosom Res. 2013;75: 160–166. doi:10.1016/j.jpsychores.2013.06.006

39. Ferreira-Valente A, Garcia IQ, Rosa AM, Pereira A, Pais-Ribeiro JL, Jensen MP. The Portuguese 35-item Survey of Pain Attitudes applied to Portuguese women with Endometriosis. Scand J Pain. 2019;19: 553–563. doi:10.1515/sjpain-2019-0004

40. Hunter M. The women’s health questionnaire: A measure of mid-aged women’s perceptions of their emotional and physical health. Psychol Health. 1992;7: 45–54. doi:10.1080/08870449208404294

41. Hunter M. The Women’s Health Questionnaire (WHQ): The development, standardization and application of a measure of mid-aged women’s emotional and physical health. Quality of Life Research. 2000;9: 733–738. doi:10.1023/A:1008973822876

42. Colantonio A, Harris JE, Tarek N. Reliability of a Health Questionnaire Among Women With Brain Injury. Journal of Neuroscience Nursing. 2011;43: 141–148. doi:10.1097/JNN.0b013e3182135b13
