# Supplementary material for: Measurement of changes to the menstrual cycle: A transdisciplinary systematic review evaluating measure quality and utility for clinical trials
Source: PLoS One. 2024 Jul 25;19(7):e0306491. doi: 10.1371/journal.pone.0306491 (PMC11271926; doi:10.1371/journal.pone.0306491)
Supplement: Attachment — Submitted filename: Response_to_Reviewer.docx [file pone.0306491.s005.docx]

**AUTHORS’ RESPONSE**

**Reviewers' Comments to Author:**

Thank you for the opportunity to review this manuscript. The authors carried out a systematic review of the instruments used to assess changes in the menstrual cycle. The subject is of interest. Many instruments are used to evaluate Abnormal/heavy menstrual bleeding, making comparisons between studies difficult. Likewise, the aspects evaluated are not always the same, creating difficulties in comparison. However, some aspects of the submitted manuscript need to be revised or better explained.

**Author response**: We thank the Reviewer for their comments to improve our manuscript and appreciate the opportunity to revise and resubmit.

1. The most relevant aspect is the length of the manuscript. The article is very long and sometimes its objectives are lost or not clear. I suggest an extensive review to make reading more straightforward. I also suggest not repeating information in the text and tables. For example, the inclusion criteria are in the text and in a table, the data extraction process is described on page 27 and in figure 1. The dates established for the review are mentioned several times.

**Author response**: In response to this helpful feedback, we have reduced the length of the manuscript by nearly 30% from over 9,000 words to less than 6,500 (including listing the inclusion criteria only in Table 2, greatly reducing the explanation of our title/abstract screening and full text review to not repeat information in Figure 1, and only mentioning the dates as an inclusion criterion and in the search terms). To ensure our search and review methodology are replicable, we have moved these details from the main paper into an Appendix in the Supporting Information. We have also sought to make our review objectives more clear through this reduction and revision (please see response to the next two comments for detail).

2. Another aspect concerns non-aligned information. In the section “ABSTRACT” the authors mentioned: “We sought to systematically review approaches to measure four aspects of changes to the menstrual cycle—bleeding, blood, pain, and perceptions— caused by any source and used in any field, however, the “Limitations” section presents another focus. It is not clear that the authors limited their review as mentioned in this statement “our primary use of the review was for the context of contraceptive clinical trials, so it is possible this internal aim may have biased our decisions about including or excluding articles.” It is a central point to consider the results obtained and accept this review. Authors need to be very clear about the objectives and methods used

**Author response**: Thank you for pointing out this opportunity to clarify our review objectives and approach, both of which are essential aspects of the paper for readers to easily understand. We have revised the “Review scope” and “Clinical trial context” sections of the Introduction and the “Limitations of the review” section of the Discussion. These edits will help to clarify that the research team’s motivations for conducting this review were for planned future work in contraceptive clinical trials, but that the intended, wider utility of the review includes clinical trials (outside of contraception), as well as menstrual health research beyond clinical trials.

3. Abstract – page 10, line 33 “... only three instruments had good scores of both quality and utility” . I suggest mentioning what these three instruments are. I suggest rewriting the conclusion.

**Author response**: We have revised the abstract to mirror the clarifying revisions taken to the text as described above. Given the lengthy names of the instruments and word count limit to the abstract, we have decided to not include the names of the three instruments, but we still wish to make the important point that we scored very few instruments as both of high quality and high utility. As for the conclusion, we think the ending sentences of the abstract are a good summary of our conclusions at the end of the paper and are not quite sure what revisions the Reviewer is seeking. If of importance, we welcome clarity on what is needed or missing from the conclusion of the abstract.

4. Languages, page 29- Why do the authors consider this section important? This information is in the table. I suggest removing.

**Author response**: We appreciate this question on instrument languages. Although these data are reported in Tables 4 and S4, we found it notable that 41% of instruments were only in English and most were only in colonial languages. Given the wider goal of working towards uniform measurement across contexts, we believe it is important to highlight current instruments are not available in the native languages of the global majority. On the positive side, it is also good to note there are a few instruments validated in multiple languages, demonstrating the possibility with future research.

5. Menstrual change(s) measured – this is a very long section. Did the authors consider presenting this information in a table and removing it from the text?

**Author response**: Thank you for the very helpful suggestion of presenting this information in a table, which we have now done in the new Table 5.

6. The section “Clinical Context” can also be shortened.

**Author response**: Thanks to the Reviewer’s comment, we can understand how this section was not well linked to the review objectives as originally written. Although we have not reduced the length (partially because of the important clarification added in response to comments above), we have revised this section so its purpose is more clearly tied to the review scope and objectives.

7. Why did the authors include tools that only assessed pain? (see table 4, e.g. Pain drawing, visual analogue scales: pain). If the main objective defined for the review was bleeding, these specific tools are not aligned with these criteria.

**Author response**: Thank you for pointing out how we can make Table 4 more clear. We have added “Uterine” before pain in the column header and the row above the listed instruments. Our review objective was to identify instruments to measure changes in menstrual bleeding patterns, menstrual blood, uterine cramping and pain, and perceptions of bleeding, blood, or pain. Because pain related to the menstrual cycle can include other types of pain (e.g., migraines, breast pain, and muscle pain), we have specified this in Table 4 and throughout the manuscript so readers can readily understand its inclusion.

8. I would like to know the authors' opinion regarding the best instruments, since many instruments only evaluate quantitative changes in the menstrual cycle, others only evaluate qualitatively (self-perception). My opinion is that an ideal instrument should evaluate both aspects, especially considering the position of the most important world societies regarding Abnormal Uterine Bleeding.

**Author response**: We thank the Reviewer for this question and see how it is relevant to those using instruments to measure menstrual changes, who would be readers of this paper. As mixed methods researchers, we also agree that an approach to assess quantitative and self-perception aspects of menstrual changes are key. We sought to address this question about instruments by evaluating the various aspects of measure quality and clinical trial utility to indicate which instruments are better (i.e., scored higher) for these attributes, which we lay out in the right columns with scores in Table 4. However, we do not believe we can identify “the best instruments” for a number of reasons. First, given the gaps in the current instrument landscape, we do not yet see sufficient evidence of validation of instruments for use to measure a variety of menstrual changes across contexts. Second, given this limitation, the best instrument for a given menstrual change—or for menstrual changes—and context will vary based on those factors, which we sought to lay out across Table 4. And third, for the clinical trial context, the FDA has specific methodological guidance on establishing evidence of Patient Reported Outcome (PRO) measures and validating such measures for a specific concept of interest and context of use. Although some of the instruments for heavy menstrual bleeding and other menstrual or gynecologic disorders and symptoms reach this threshold, none do for the broad menstruating population for the range of menstrual changes likely to occur across clinical trials and related research.

9. I disagree that “the exclusion of other types of uterine bleeding outside the menstrual cycle, such as bleeding during pregnancy, during breastfeeding and after menopause” may be a limitation of your study, as presented. If the authors clearly define the objectives and define the exclusion of these situations (inclusion and exclusion criteria), they do not need to point out this limitation

**Author response**: We agree with the Reviewer and have revised this sentence in the “Limitations” section of the Discussion.
