# Supplementary material for: Measurement of changes to the menstrual cycle: A transdisciplinary systematic review evaluating measure quality and utility for clinical trials
Source: PLoS One. 2024 Jul 25;19(7):e0306491. doi: 10.1371/journal.pone.0306491 (PMC11271926; doi:10.1371/journal.pone.0306491)
Supplement: Peer review history

# Peer review: Decision Letter 0

**DOI:** 10.1371/journal.pone.0306491.r001

**Authors:** Avanti Dey

10 Apr 2024

PONE-D-24-02620Measurement of changes to the menstrual cycle: a transdisciplinary systematic review evaluating measure quality and utility for clinical trialsPLOS ONE

Dear Dr. Mackenzie,

Thank you for submitting your manuscript to PLOS ONE. After careful consideration, we feel that it has merit but does not fully meet PLOS ONE’s publication criteria as it currently stands. Therefore, we invite you to submit a revised version of the manuscript that addresses the points raised during the review process.

Please note that we have only been able to secure a single reviewer to assess your manuscript. We are issuing a decision on your manuscript at this point to prevent further delays in the evaluation of your manuscript. Please be aware that the editor who handles your revised manuscript might find it necessary to invite additional reviewers to assess this work once the revised manuscript is submitted. However, we will aim to proceed on the basis of this single review if possible. 

Please submit your revised manuscript by May 25 2024 11:59PM. If you will need more time than this to complete your revisions, please reply to this message or contact the journal office at plosone@plos.org. When you're ready to submit your revision, log on to https://www.editorialmanager.com/pone/ and select the 'Submissions Needing Revision' folder to locate your manuscript file.

Please include the following items when submitting your revised manuscript:

- A rebuttal letter that responds to each point raised by the academic editor and reviewer(s). You should upload this letter as a separate file labeled 'Response to Reviewers'.
- A marked-up copy of your manuscript that highlights changes made to the original version. You should upload this as a separate file labeled 'Revised Manuscript with Track Changes'.
- An unmarked version of your revised paper without tracked changes. You should upload this as a separate file labeled 'Manuscript'.

If you would like to make changes to your financial disclosure, please include your updated statement in your cover letter. Guidelines for resubmitting your figure files are available below the reviewer comments at the end of this letter.

If applicable, we recommend that you deposit your laboratory protocols in protocols.io to enhance the reproducibility of your results. Protocols.io assigns your protocol its own identifier (DOI) so that it can be cited independently in the future. For instructions see: https://journals.plos.org/plosone/s/submission-guidelines#loc-laboratory-protocols. Additionally, PLOS ONE offers an option for publishing peer-reviewed Lab Protocol articles, which describe protocols hosted on protocols.io. Read more information on sharing protocols at https://plos.org/protocols?utm_medium=editorial-email&utm_source=authorletters&utm_campaign=protocols.

We look forward to receiving your revised manuscript.

Kind regards,

Avanti Dey, PhD

Staff Editor

PLOS ONE

Journal Requirements:

Additional Editor Comments (if provided):

[Note: HTML markup is below. Please do not edit.]

Reviewers' comments:

Reviewer's Responses to Questions

**Comments to the Author**

1. Is the manuscript technically sound, and do the data support the conclusions?

The manuscript must describe a technically sound piece of scientific research with data that supports the conclusions. Experiments must have been conducted rigorously, with appropriate controls, replication, and sample sizes. The conclusions must be drawn appropriately based on the data presented. 

Reviewer #1: Yes

**********

2. Has the statistical analysis been performed appropriately and rigorously? 

Reviewer #1: N/A

**********

3. Have the authors made all data underlying the findings in their manuscript fully available?

The PLOS Data policy requires authors to make all data underlying the findings described in their manuscript fully available without restriction, with rare exception (please refer to the Data Availability Statement in the manuscript PDF file). The data should be provided as part of the manuscript or its supporting information, or deposited to a public repository. For example, in addition to summary statistics, the data points behind means, medians and variance measures should be available. If there are restrictions on publicly sharing data—e.g. participant privacy or use of data from a third party—those must be specified.

Reviewer #1: Yes

**********

4. Is the manuscript presented in an intelligible fashion and written in standard English?

PLOS ONE does not copyedit accepted manuscripts, so the language in submitted articles must be clear, correct, and unambiguous. Any typographical or grammatical errors should be corrected at revision, so please note any specific errors here.

Reviewer #1: No

**********

5. Review Comments to the Author

Please use the space provided to explain your answers to the questions above. You may also include additional comments for the author, including concerns about dual publication, research ethics, or publication ethics. (Please upload your review as an attachment if it exceeds 20,000 characters)

Reviewer #1: Thank you for the opportunity to review this manuscript. The authors carried out a systematic review of the instruments used to assess changes in the menstrual cycle. The subject is of interest. Many instruments are used to evaluate Abnormal/heavy menstrual bleeding, making comparisons between studies difficult. Likewise, the aspects evaluated are not always the same, creating difficulties in comparison. However, some aspects of the submitted manuscript need to be revised or better explained.

The most relevant aspect is the length of the manuscript. The article is very long and sometimes its objectives are lost or not clear. I suggest an extensive review to make reading more straightforward. I also suggest not repeating information in the text and tables. For example, the inclusion criteria are in the text and in a table, the data extraction process is described on page 27 and in figure 1. The dates established for the review are mentioned several times.

Another aspect concerns non-aligned information. In the section “ABSTRACT” the authors mentioned: “We sought to systematically review approaches to measure four aspects of changes to the menstrual cycle—bleeding, blood, pain, and perceptions— caused by any source and used in any field, however, the “Limitations” section presents another focus. It is not clear that the authors limited their review as mentioned in this statement “our primary use of the review was for the context of contraceptive clinical trials, so it is possible this internal aim may have biased our decisions about including or excluding articles.” It is a central point to consider the results obtained and accept this review. Authors need to be very clear about the objectives and methods used

My other considerations:

Abstract – page 10, line 33 “... only three instruments had good scores of both quality and utility” . I suggest mentioning what these three instruments are. I suggest rewriting the conclusion.

Languages, page 29- Why do the authors consider this section important? This information is in the table. I suggest removing.

Menstrual change(s) measured – this is a very long section. Did the authors consider presenting this information in a table and removing it from the text?

The section “Clinical Context” can also be shortened.

Why did the authors include tools that only assessed pain? (see table 4, e.g. Pain drawing, visual analogue scales: pain). If the main objective defined for the review was bleeding, these specific tools are not aligned with these criteria.

I would like to know the authors' opinion regarding the best instruments, since many instruments only evaluate quantitative changes in the menstrual cycle, others only evaluate qualitatively (self-perception). My opinion is that an ideal instrument should evaluate both aspects, especially considering the position of the most important world societies regarding Abnormal Uterine Bleeding.

I disagree that “the exclusion of other types of uterine bleeding outside the menstrual cycle, such as bleeding during pregnancy, during breastfeeding and after menopause” may be a limitation of your study, as presented. If the authors clearly define the objectives and define the exclusion of these situations (inclusion and exclusion criteria), they do not need to point out this limitation

**********

6. PLOS authors have the option to publish the peer review history of their article (what does this mean?). If published, this will include your full peer review and any attached files.

If you choose “no”, your identity will remain anonymous but your review may still be made public.

**Do you want your identity to be public for this peer review?** For information about this choice, including consent withdrawal, please see our Privacy Policy.

Reviewer #1: No

**********

[NOTE: If reviewer comments were submitted as an attachment file, they will be attached to this email and accessible via the submission site. Please log into your account, locate the manuscript record, and check for the action link "View Attachments". If this link does not appear, there are no attachment files.]

While revising your submission, please upload your figure files to the Preflight Analysis and Conversion Engine (PACE) digital diagnostic tool, https://pacev2.apexcovantage.com/. PACE helps ensure that figures meet PLOS requirements. To use PACE, you must first register as a user. Registration is free. Then, login and navigate to the UPLOAD tab, where you will find detailed instructions on how to use the tool. If you encounter any issues or have any questions when using PACE, please email PLOS at figures@plos.org. Please note that Supporting Information files do not need this step.

# Author comment: Author response to Decision Letter 0

**DOI:** 10.1371/journal.pone.0306491.r002

1 Jun 2024

AUTHORS’ RESPONSE

Reviewers' Comments to Author: 

Thank you for the opportunity to review this manuscript. The authors carried out a systematic review of the instruments used to assess changes in the menstrual cycle. The subject is of interest. Many instruments are used to evaluate Abnormal/heavy menstrual bleeding, making comparisons between studies difficult. Likewise, the aspects evaluated are not always the same, creating difficulties in comparison. However, some aspects of the submitted manuscript need to be revised or better explained.

Author response: We thank the Reviewer for their comments to improve our manuscript and appreciate the opportunity to revise and resubmit.

1. The most relevant aspect is the length of the manuscript. The article is very long and sometimes its objectives are lost or not clear. I suggest an extensive review to make reading more straightforward. I also suggest not repeating information in the text and tables. For example, the inclusion criteria are in the text and in a table, the data extraction process is described on page 27 and in figure 1. The dates established for the review are mentioned several times.

Author response: In response to this helpful feedback, we have reduced the length of the manuscript by nearly 30% from over 9,000 words to less than 6,500 (including listing the inclusion criteria only in Table 2, greatly reducing the explanation of our title/abstract screening and full text review to not repeat information in Figure 1, and only mentioning the dates as an inclusion criterion and in the search terms). To ensure our search and review methodology are replicable, we have moved these details from the main paper into an Appendix in the Supporting Information. We have also sought to make our review objectives more clear through this reduction and revision (please see response to the next two comments for detail).

2. Another aspect concerns non-aligned information. In the section “ABSTRACT” the authors mentioned: “We sought to systematically review approaches to measure four aspects of changes to the menstrual cycle—bleeding, blood, pain, and perceptions— caused by any source and used in any field, however, the “Limitations” section presents another focus. It is not clear that the authors limited their review as mentioned in this statement “our primary use of the review was for the context of contraceptive clinical trials, so it is possible this internal aim may have biased our decisions about including or excluding articles.” It is a central point to consider the results obtained and accept this review. Authors need to be very clear about the objectives and methods used

Author response: Thank you for pointing out this opportunity to clarify our review objectives and approach, both of which are essential aspects of the paper for readers to easily understand. We have revised the “Review scope” and “Clinical trial context” sections of the Introduction and the “Limitations of the review” section of the Discussion. These edits will help to clarify that the research team’s motivations for conducting this review were for planned future work in contraceptive clinical trials, but that the intended, wider utility of the review includes clinical trials (outside of contraception), as well as menstrual health research beyond clinical trials.

3. Abstract – page 10, line 33 “... only three instruments had good scores of both quality and utility” . I suggest mentioning what these three instruments are. I suggest rewriting the conclusion.

Author response: We have revised the abstract to mirror the clarifying revisions taken to the text as described above. Given the lengthy names of the instruments and word count limit to the abstract, we have decided to not include the names of the three instruments, but we still wish to make the important point that we scored very few instruments as both of high quality and high utility. As for the conclusion, we think the ending sentences of the abstract are a good summary of our conclusions at the end of the paper and are not quite sure what revisions the Reviewer is seeking. If of importance, we welcome clarity on what is needed or missing from the conclusion of the abstract. 

4. Languages, page 29- Why do the authors consider this section important? This information is in the table. I suggest removing.

Author response: We appreciate this question on instrument languages. Although these data are reported in Tables 4 and S4, we found it notable that 41% of instruments were only in English and most were only in colonial languages. Given the wider goal of working towards uniform measurement across contexts, we believe it is important to highlight current instruments are not available in the native languages of the global majority. On the positive side, it is also good to note there are a few instruments validated in multiple languages, demonstrating the possibility with future research. 

5. Menstrual change(s) measured – this is a very long section. Did the authors consider presenting this information in a table and removing it from the text?

Author response: Thank you for the very helpful suggestion of presenting this information in a table, which we have now done in the new Table 5.

6. The section “Clinical Context” can also be shortened.

Author response: Thanks to the Reviewer’s comment, we can understand how this section was not well linked to the review objectives as originally written. Although we have not reduced the length (partially because of the important clarification added in response to comments above), we have revised this section so its purpose is more clearly tied to the review scope and objectives.

7. Why did the authors include tools that only assessed pain? (see table 4, e.g. Pain drawing, visual analogue scales: pain). If the main objective defined for the review was bleeding, these specific tools are not aligned with these criteria.

Author response: Thank you for pointing out how we can make Table 4 more clear. We have added “Uterine” before pain in the column header and the row above the listed instruments. Our review objective was to identify instruments to measure changes in menstrual bleeding patterns, menstrual blood, uterine cramping and pain, and perceptions of bleeding, blood, or pain. Because pain related to the menstrual cycle can include other types of pain (e.g., migraines, breast pain, and muscle pain), we have specified this in Table 4 and throughout the manuscript so readers can readily understand its inclusion.

8. I would like to know the authors' opinion regarding the best instruments, since many instruments only evaluate quantitative changes in the menstrual cycle, others only evaluate qualitatively (self-perception). My opinion is that an ideal instrument should evaluate both aspects, especially considering the position of the most important world societies regarding Abnormal Uterine Bleeding.

Author response: We thank the Reviewer for this question and see how it is relevant to those using instruments to measure menstrual changes, who would be readers of this paper. As mixed methods researchers, we also agree that an approach to assess quantitative and self-perception aspects of menstrual changes are key. We sought to address this question about instruments by evaluating the various aspects of measure quality and clinical trial utility to indicate which instruments are better (i.e., scored higher) for these attributes, which we lay out in the right columns with scores in Table 4. However, we do not believe we can identify “the best instruments” for a number of reasons. First, given the gaps in the current instrument landscape, we do not yet see sufficient evidence of validation of instruments for use to measure a variety of menstrual changes across contexts. Second, given this limitation, the best instrument for a given menstrual change—or for menstrual changes—and context will vary based on those factors, which we sought to lay out across Table 4. And third, for the clinical trial context, the FDA has specific methodological guidance on establishing evidence of Patient Reported Outcome (PRO) measures and validating such measures for a specific concept of interest and context of use. Although some of the instruments for heavy menstrual bleeding and other menstrual or gynecologic disorders and symptoms reach this threshold, none do for the broad menstruating population for the range of menstrual changes likely to occur across clinical trials and related research. 

9. I disagree that “the exclusion of other types of uterine bleeding outside the menstrual cycle, such as bleeding during pregnancy, during breastfeeding and after menopause” may be a limitation of your study, as presented. If the authors clearly define the objectives and define the exclusion of these situations (inclusion and exclusion criteria), they do not need to point out this limitation

Author response: We agree with the Reviewer and have revised this sentence in the “Limitations” section of the Discussion.

Attachment Submitted filename: Response_to_Reviewer.docx

# Peer review: Decision Letter 1

**DOI:** 10.1371/journal.pone.0306491.r003

**Authors:** Alison Parker

19 Jun 2024

Measurement of changes to the menstrual cycle: A transdisciplinary systematic review evaluating measure quality and utility for clinical trials

PONE-D-24-02620R1

Dear Dr. Mackenzie,

We’re pleased to inform you that your manuscript has been judged scientifically suitable for publication and will be formally accepted for publication once it meets all outstanding technical requirements.

Within one week, you’ll receive an e-mail detailing the required amendments. When these have been addressed, you’ll receive a formal acceptance letter and your manuscript will be scheduled for publication.

An invoice will be generated when your article is formally accepted. Please note, if your institution has a publishing partnership with PLOS and your article meets the relevant criteria, all or part of your publication costs will be covered. Please make sure your user information is up-to-date by logging into Editorial Manager at Editorial Manager® and clicking the ‘Update My Information' link at the top of the page. If you have any questions relating to publication charges, please contact our Author Billing department directly at authorbilling@plos.org.

If your institution or institutions have a press office, please notify them about your upcoming paper to help maximize its impact. If they’ll be preparing press materials, please inform our press team as soon as possible -- no later than 48 hours after receiving the formal acceptance. Your manuscript will remain under strict press embargo until 2 pm Eastern Time on the date of publication. For more information, please contact onepress@plos.org.

Kind regards,

Alison Parker

Academic Editor

PLOS ONE

Additional Editor Comments (optional):

Reviewers' comments:

Reviewer's Responses to Questions

**Comments to the Author**

1. If the authors have adequately addressed your comments raised in a previous round of review and you feel that this manuscript is now acceptable for publication, you may indicate that here to bypass the “Comments to the Author” section, enter your conflict of interest statement in the “Confidential to Editor” section, and submit your "Accept" recommendation.

Reviewer #1: All comments have been addressed

**********

2. Is the manuscript technically sound, and do the data support the conclusions?

The manuscript must describe a technically sound piece of scientific research with data that supports the conclusions. Experiments must have been conducted rigorously, with appropriate controls, replication, and sample sizes. The conclusions must be drawn appropriately based on the data presented. 

Reviewer #1: Yes

**********

3. Has the statistical analysis been performed appropriately and rigorously? 

Reviewer #1: N/A

**********

4. Have the authors made all data underlying the findings in their manuscript fully available?

The PLOS Data policy requires authors to make all data underlying the findings described in their manuscript fully available without restriction, with rare exception (please refer to the Data Availability Statement in the manuscript PDF file). The data should be provided as part of the manuscript or its supporting information, or deposited to a public repository. For example, in addition to summary statistics, the data points behind means, medians and variance measures should be available. If there are restrictions on publicly sharing data—e.g. participant privacy or use of data from a third party—those must be specified.

Reviewer #1: Yes

**********

5. Is the manuscript presented in an intelligible fashion and written in standard English?

PLOS ONE does not copyedit accepted manuscripts, so the language in submitted articles must be clear, correct, and unambiguous. Any typographical or grammatical errors should be corrected at revision, so please note any specific errors here.

Reviewer #1: Yes

**********

6. Review Comments to the Author

Please use the space provided to explain your answers to the questions above. You may also include additional comments for the author, including concerns about dual publication, research ethics, or publication ethics. (Please upload your review as an attachment if it exceeds 20,000 characters)

Reviewer #1: The authors included the reviewer's suggestions in the current version of the manuscript and they answered all questions.

**********

7. PLOS authors have the option to publish the peer review history of their article (what does this mean?). If published, this will include your full peer review and any attached files.

If you choose “no”, your identity will remain anonymous but your review may still be made public.

**Do you want your identity to be public for this peer review?** For information about this choice, including consent withdrawal, please see our Privacy Policy.

Reviewer #1: No

**********

# Editor report: Acceptance letter

**DOI:** 10.1371/journal.pone.0306491.r004

**Authors:** Alison Parker

16 Jul 2024

PONE-D-24-02620R1 

PLOS ONE

Dear Dr. Mackenzie, 

I'm pleased to inform you that your manuscript has been deemed suitable for publication in PLOS ONE. Congratulations! Your manuscript is now being handed over to our production team.

At this stage, our production department will prepare your paper for publication. This includes ensuring the following:

* All references, tables, and figures are properly cited

* All relevant supporting information is included in the manuscript submission,

* There are no issues that prevent the paper from being properly typeset

If revisions are needed, the production department will contact you directly to resolve them. If no revisions are needed, you will receive an email when the publication date has been set. At this time, we do not offer pre-publication proofs to authors during production of the accepted work. Please keep in mind that we are working through a large volume of accepted articles, so please give us a few weeks to review your paper and let you know the next and final steps. 

Lastly, if your institution or institutions have a press office, please let them know about your upcoming paper now to help maximize its impact. If they'll be preparing press materials, please inform our press team within the next 48 hours. Your manuscript will remain under strict press embargo until 2 pm Eastern Time on the date of publication. For more information, please contact onepress@plos.org.

If we can help with anything else, please email us at customercare@plos.org.

Thank you for submitting your work to PLOS ONE and supporting open access. 

Kind regards, 

PLOS ONE Editorial Office Staff

on behalf of

Dr. Alison Parker 

Academic Editor

PLOS ONE
